# Age at first birth in women is genetically associated with increased risk of schizophrenia

**DOI:** 10.1038/s41598-018-28160-z

**Published:** 2018-07-05

**Authors:** Guiyan Ni, Jacob Gratten, Naomi R. Wray, Sang Hong Lee, Schizophrenia Working Group of the Psychiatric Genomics Consortium, Stephan Ripke, Benjamin M. Neale, Aiden Corvin, James T. R. Walters, Kai-How Farh, Peter A. Holmans, Phil Lee, Brendan Bulik-Sullivan, David A. Collier, Hailiang Huang, Tune H. Pers, Ingrid Agartz, Esben Agerbo, Margot Albus, Madeline Alexander, Farooq Amin, Silviu A. Bacanu, Martin Begemann, Richard A. Belliveau, Judit Bene, Sarah E. Bergen, Elizabeth Bevilacqua, Tim B. Bigdeli, Donald W. Black, Richard Bruggeman, Nancy G. Buccola, Randy L. Buckner, William Byerley, Wiepke Cahn, Guiqing Cai, Dominique Campion, Rita M. Cantor, Vaughan J. Carr, Noa Carrera, Stanley V. Catts, Kimberly D. Chambert, Raymond C. K. Chan, Ronald Y. L. Chen, Eric Y. H. Chen, Wei Cheng, Eric F. C. Cheung, Siow Ann Chong, C. Robert Cloninger, David Cohen, Nadine Cohen, Paul Cormican, Nick Craddock, James J. Crowley, David Curtis, Michael Davidson, Kenneth L. Davis, Franziska Degenhardt, Jurgen Del Favero, Ditte Demontis, Dimitris Dikeos, Timothy Dinan, Srdjan Djurovic, Gary Donohoe, Elodie Drapeau, Jubao Duan, Frank Dudbridge, Naser Durmishi, Peter Eichhammer, Johan Eriksson, Valentina Escott-Price, Laurent Essioux, Ayman H. Fanous, Martilias S. Farrell, Josef Frank, Lude Franke, Robert Freedman, Nelson B. Freimer, Marion Friedl, Joseph I. Friedman, Menachem Fromer, Giulio Genovese, Lyudmila Georgieva, Ina Giegling, Paola Giusti-Rodríguez, Stephanie Godard, Jacqueline I. Goldstein, Vera Golimbet, Srihari Gopal, Lieuwe de Haan, Christian Hammer, Marian L. Hamshere, Mark Hansen, Thomas Hansen, Vahram Haroutunian, Annette M. Hartmann, Frans A. Henskens, Stefan Herms, Joel N. Hirschhorn, Per Hoffmann, Andrea Hofman, Mads V. Hollegaard, David M. Hougaard, Masashi Ikeda, Inge Joa, Antonio Juliá, René S. Kahn, Luba Kalaydjieva, Sena Karachanak-Yankova, Juha Karjalainen, David Kavanagh, Matthew C. Keller, James L. Kennedy, Andrey Khrunin, Yunjung Kim, Janis Klovins, James A. Knowles, Bettina Konte, Vaidutis Kucinskas, Zita Ausrele Kucinskiene, Hana Kuzelova-Ptackova, Anna K. Kähler, Claudine Laurent, Jimmy Lee Chee Keong, Sophie E. Legge, Bernard Lerer, Miaoxin Li, Tao Li, Kung-Yee Liang, Jeffrey Lieberman, Svetlana Limborska, Carmel M. Loughland, Jan Lubinski, Jouko Lönnqvist, Milan Macek, Patrik K. E. Magnusson, Brion S. Maher, Wolfgang Maier, Jacques Mallet, Sara Marsal, Manuel Mattheisen, Morten Mattingsdal, Robert W. McCarley, Colm McDonald, Andrew M. McIntosh, Sandra Meier, Carin J. Meijer, Bela Melegh, Ingrid Melle, Raquelle I. Mesholam-Gately, Andres Metspalu, Patricia T. Michie, Lili Milani, Vihra Milanova, Younes Mokrab, Derek W. Morris, Ole Mors, Kieran C. Murphy, Robin M. Murray, Inez Myin-Germeys, Bertram Müller-Myhsok, Mari Nelis, Igor Nenadic, Deborah A. Nertney, Gerald Nestadt, Kristin K. Nicodemus, Liene Nikitina-Zake, Laura Nisenbaum, Annelie Nordin, Eadbhard O’Callaghan, Colm O’Dushlaine, F. Anthony O’Neill, Sang-Yun Oh, Ann Olincy, Line Olsen, Jim Van Os, Christos Pantelis, George N. Papadimitriou, Sergi Papiol, Elena Parkhomenko, Michele T. Pato, Tiina Paunio, Milica Pejovic-Milovancevic, Diana O. Perkins, Olli Pietiläinen, Jonathan Pimm, Andrew J. Pocklington, John Powell, Alkes Price, Ann E. Pulver, Shaun M. Purcell, Digby Quested, Henrik B. Rasmussen, Abraham Reichenberg, Mark A. Reimers, Alexander L. Richards, Joshua L. Roffman, Panos Roussos, Douglas M. Ruderfer, Veikko Salomaa, Alan R. Sanders, Ulrich Schall, Christian R. Schubert, Thomas G. Schulze, Sibylle G. Schwab, Edward M. Scolnick, Rodney J. Scott, Larry J. Seidman, Jianxin Shi, Engilbert Sigurdsson, Teimuraz Silagadze, Jeremy M. Silverman, Kang Sim, Petr Slominsky, Jordan W. Smoller, Hon-Cheong So, Chris C. A. Spencer, Eli A. Stahl, Hreinn Stefansson, Stacy Steinberg, Elisabeth Stogmann, Richard E. Straub, Eric Strengman, Jana Strohmaier, T. Scott Stroup, Mythily Subramaniam, Jaana Suvisaari, Dragan M. Svrakic, Jin P. Szatkiewicz, Erik Söderman, Srinivas Thirumalai, Draga Toncheva, Sarah Tosato, Juha Veijola, John Waddington, Dermot Walsh, Dai Wang, Qiang Wang, Bradley T. Webb, Mark Weiser, Dieter B. Wildenauer, Nigel M. Williams, Stephanie Williams, Stephanie H. Witt, Aaron R. Wolen, Emily H. M. Wong, Brandon K. Wormley, Hualin Simon Xi, Clement C. Zai, Xuebin Zheng, Fritz Zimprich, Kari Stefansson, Peter M. Visscher, Rolf Adolfsson, Ole A. Andreassen, Douglas H. R. Blackwood, Elvira Bramon, Joseph D. Buxbaum, Anders D. Børglum, Sven Cichon, Ariel Darvasi, Enrico Domenici, Hannelore Ehrenreich, Tõnu Esko, Pablo V. Gejman, Michael Gill, Hugh Gurling, Christina M. Hultman, Nakao Iwata, Assen V. Jablensky, Erik G. Jönsson, Kenneth S. Kendler, George Kirov, Jo Knight, Todd Lencz, Douglas F. Levinson, Qingqin S. Li, Jianjun Liu, Anil K. Malhotra, Steven A. McCarroll, Andrew McQuillin, Jennifer L. Moran, Preben B. Mortensen, Bryan J. Mowry, Markus M. Nöthen, Roel A. Ophoff, Michael J. Owen, Aarno Palotie, Carlos N. Pato, Tracey L. Petryshen, Danielle Posthuma, Marcella Rietschel, Brien P. Riley, Dan Rujescu, Pak C. Sham, Pamela Sklar, David St. Clair, Daniel R. Weinberger, Jens R. Wendland, Thomas Werge, Mark J. Daly, Patrick F. Sullivan, Michael C. O’Donovan

**Affiliations:** 1 Australian Center for Precision Health, University of South Australia Cancer Research Institute, University of South Australia, Adelaide, SA 5000 Australia; 2 School of Environmental and Rural Science, University of New England, Armidale, NSW 2351 Australia; 3 Institute for Molecular Bioscience, University of Queensland, Brisbane, Queensland 4072 Australia; 4 Queensland Brain Institute, University of Queensland, Brisbane, Queensland 4072 Australia; 5 Analytic and Translational Genetics Unit, Massachusetts General Hospital, Boston, Massachusetts 02114 USA; 6 Stanley Center for Psychiatric Research, Broad Institute of MIT and Harvard, Cambridge, Massachusetts 02142 USA; 7 Medical and Population Genetics Program, Broad Institute of MIT and Harvard, Cambridge, Massachusetts 02142 USA; 8 Psychiatric and Neurodevelopmental Genetics Unit, Massachusetts General Hospital, Boston, Massachusetts 02114 USA; 9 Neuropsychiatric Genetics Research Group, Department of Psychiatry, Trinity College Dublin, Dublin, 8 Ireland; 10 MRC Centre for Neuropsychiatric Genetics and Genomics, Institute of Psychological Medicine and Clinical Neurosciences, School of Medicine, Cardiff University, Cardiff, CF24 4HQ UK; 11 National Centre for Mental Health, Cardiff University, Cardiff, CF24 4HQ UK; 12 Eli Lilly and Company Limited, Erl Wood Manor, Sunninghill Road, Windlesham, Surrey GU20 6PH UK; 13 Social, Genetic and Developmental Psychiatry Centre, Institute of Psychiatry, King’s College London, London, SE5 8AF UK; 14 Center for Biological Sequence Analysis, Department of Systems Biology, Technical University of Denmark, Lyngby, DK-2800 Denmark; 15 Division of Endocrinology and Center for Basic and Translational Obesity Research, Boston Children’s Hospital, Boston, Massachusetts, 02115 USA; 16 Department of Clinical Neuroscience, Psychiatry Section, Karolinska Institutet, SE 17176 Stockholm, Sweden; 17 Department of Psychiatry, Diakonhjemmet Hospital, 0319 Oslo, Norway; 18 NORMENT, KG Jebsen Centre for Psychosis Research, Institute of Clinical Medicine, University of Oslo, 0424 Oslo, Norway; 19 Centre for Integrative Register-based Research, CIRRAU, Aarhus University, DK 8210 Aarhus, Denmark; 20 National Centre for Register-based Research, AarhusUniversity, DK 8210 Aarhus, Denmark; 21 The Lundbeck Foundation Initiative for Integrative Psychiatric Research, iPSYCH, Aarhus, Denmark; 22 State Mental Hospital, 85540 Haar, Germany; 23 Department of Psychiatry and Behavioral Sciences, Stanford University, Stanford, California 94305 USA; 24 Department of Psychiatry and Behavioral Sciences, Atlanta Veterans Affairs Medical Center, Atlanta, Georgia 30033 USA; 25 Department of Psychiatry and Behavioral Sciences, Emory University, Atlanta, Georgia 30322 USA; 26 Virginia Institute for Psychiatric and Behavioral Genetics, Department of Psychiatry, Virginia Commonwealth University, Richmond, Virginia 23298 USA; 27 Clinical Neuroscience, Max Planck Institute of Experimental Medicine, Goettingen, 37075 Germany; 28 Department of Medical Genetics, University of Pécs, Pécs, H-7624 Hungary; 29 Szentagothai Research Center, University of Pécs, Pécs, H-7624 Hungary; 30 Department of Medical Epidemiology and Biostatistics, Karolinska Institutet, Stockholm, SE-17177 Sweden; 31 Department of Psychiatry, University of Iowa Carver College of Medicine, Iowa City, Iowa 52242 USA; 32 University Medical Center Groningen, Department of Psychiatry, University of Groningen, Groningen, NL-9700 RB The Netherlands; 33 School of Nursing, Louisiana State University Health Sciences Center, New Orleans, Louisiana 70112 USA; 34 Athinoula A Martinos Center, Massachusetts General Hospital, Boston, Massachusetts 02129 USA; 35 Center for Brain Science, Harvard University, Cambridge, Massachusetts 02138 USA; 36 Department of Psychiatry, Massachusetts General Hospital, Boston, Massachusetts 02114 USA; 37 Department of Psychiatry, University of California at San Francisco, San Francisco, California 94143 USA; 38 University Medical Center Utrecht, Department of Psychiatry, Rudolf Magnus Institute of Neuroscience, 3584 Utrecht, The Netherlands; 39 Department of Human Genetics, Icahn School of Medicine at Mount Sinai, New York, New York 10029 USA; 40 Department of Psychiatry, Icahn School of Medicine at Mount Sinai, New York, New York 10029 USA; 41 Centre Hospitalier du Rouvray and INSERM U1079 Faculty of Medicine, 76301 Rouen, France; 42 Department of Human Genetics, David Geffen School of Medicine, University of California, Los Angeles, California 90095 USA; 43 Schizophrenia Research Institute, Sydney, NSW 2010 Australia; 44 School of Psychiatry, University of New South Wales, Sydney, NSW 2031 Australia; 45 Royal Brisbane and Women’s Hospital, University of Queensland, Brisbane, St Lucia, QLD 4072 Australia; 46 Institute of Psychology, Chinese Academy of Science, Beijing, 100101 China; 47 Department of Psychiatry, Li Ka Shing Faculty of Medicine, The University of Hong Kong, Hong Kong, China; 48 State Key Laboratory for Brain and Cognitive Sciences, Li Ka Shing Faculty of Medicine, The University of Hong Kong, Hong Kong, China; 49 Department of Computer Science, University of North Carolina, Chapel Hill, North Carolina 27514 USA; 50 Castle Peak Hospital, Hong Kong, China; 51 Institute of Mental Health, Singapore, 539747 Singapore; 52 Department of Psychiatry, Washington University, St. Louis, Missouri 63110 USA; 53 Department of Child and Adolescent Psychiatry, Assistance Publique Hopitaux de Paris, Pierre and Marie Curie Faculty of Medicine and Institute for Intelligent Systems and Robotics, Paris, 75013 France; 54 Blue Note Biosciences, Princeton, New Jersey 08540 USA; 55 Department of Genetics, University of North Carolina, Chapel Hill, North Carolina 27599-7264 USA; 56 Department of Psychological Medicine, Queen Mary University of London, London, E1 1BB UK; 57 Molecular Psychiatry Laboratory, Division of Psychiatry, University College London, London, WC1E6JJ UK; 58 Sheba Medical Center, Tel Hashomer, 52621 Israel; 59 Department of Genomics, Life and Brain Center, D-53127 Bonn, Germany; 60 Institute of Human Genetics, University of Bonn, D-53127 Bonn, Germany; 61 Applied Molecular Genomics Unit, VIB Department of Molecular Genetics, University of Antwerp, B-2610 Antwerp, Belgium; 62 Centre for Integrative Sequencing, iSEQ, Aarhus University, DK-8000 Aarhus C, Denmark; 63 Department of Biomedicine, , Aarhus University, DK-8000 Aarhus C, Denmark; 64 First Department of Psychiatry, University of Athens Medical School, Athens, 11528 Greece; 65 Department of Psychiatry, , University College Cork, Co Cork, Ireland; 66 Department of Medical Genetics, Oslo University Hospital, 0424 Oslo, Norway; 67 Cognitive Genetics and Therapy Group, School of Psychology and Discipline of Biochemistry, National University of Ireland Galway, Co Galway, Ireland; 68 Department of Psychiatry and Behavioral Neuroscience, University of Chicago, Chicago, Illinois 60637 USA; 69 Department of Psychiatry and Behavioral Sciences, NorthShore University HealthSystem, Evanston, Illinois 60201 USA; 70 Department of Non-Communicable Disease Epidemiology, London School of Hygiene and Tropical Medicine, London, WC1E 7HT UK; 71 Department of Child and Adolescent Psychiatry, University Clinic of Psychiatry, Skopje, 1000 Macedonia; 72 Department of Psychiatry, University of Regensburg, 93053 Regensburg, Germany; 73 Department of General Practice, Helsinki University Central Hospital, University of Helsinki, Po Box 20, Tukholmankatu 8 B, FI-00014 Helsinki, Finland; 74 Folkhälsan Research Center, Biomedicum Helsinki 1, Haartmaninkatu 8, FI-00290 Helsinki, Finland; 75 National Institute for Health and Welfare, PO Box 30, FI-00271 Helsinki, Finland; 76 Translational Technologies and Bioinformatics, Pharma Research and Early Development, F Hoffman-La Roche, CH-4070 Basel, Switzerland; 77 Department of Psychiatry, Georgetown University School of Medicine, Washington, DC 20057 USA; 78 Department of Psychiatry, Keck School of Medicine of the University of Southern California, Los Angeles, California 90033 USA; 79 Department of Psychiatry, Virginia Commonwealth University School of Medicine, Richmond, Virginia 23298 USA; 80 Mental Health Service Line, Washington VA Medical Center, Washington, DC 20422 USA; 81 Department of Genetic Epidemiology in Psychiatry, Central Institute of Mental Health, Medical Faculty Mannheim, University of Heidelberg, Heidelberg, D-68159 Mannheim Germany; 82 Department of Genetics, University of Groningen, University Medical Centre Groningen, 9700 RB Groningen, The Netherlands; 83 Department of Psychiatry, University of Colorado Denver, Aurora, Colorado 80045 USA; 84 Center for Neurobehavioral Genetics, Semel Institute for Neuroscience and Human Behavior, University of California, Los Angeles, California 90095 USA; 85 Department of Psychiatry, University of Halle, 06112 Halle, Germany; 86 Division of Psychiatric Genomics, Department of Psychiatry, Icahn School of Medicine at Mount Sinai, New York, New York, New York 10029 USA; 87 Department of Psychiatry, University of Munich, 80336 Munich, Germany; 88 Departments of Psychiatry and Human and Molecular Genetics, INSERM, Institut de Myologie, Hôpital de la Pitiè-Salpêtrière, Paris, 75013 France; 89 Mental Health Research Centre, Russian Academy of Medical Sciences, 115522 Moscow, Russia; 90 Neuroscience Therapeutic Area, Janssen Research and Development, Raritan, New Jersey 08869 USA; 91 Academic Medical Centre University of Amsterdam, Department of Psychiatry, 1105 AZ Amsterdam, The Netherlands; 92 Illumina, La Jolla, California, California 92122 USA; 93 Institute of Biological Psychiatry, Mental Health Centre Sct Hans, Mental Health Services Copenhagen, Copenhagen, DK-4000 Denmark; 94 Friedman Brain Institute, Icahn School of Medicine at Mount Sinai, New York New York, 10029 USA; 95 J J Peters VA Medical Center, Bronx, New York, New York 10468 USA; 96 Priority Research Centre for Health Behaviour, University of Newcastle, Newcastle, NSW 2308 Australia; 97 School of Electrical Engineering and Computer Science, University of Newcastle, Newcastle, NSW 2308 Australia; 98 Division of Medical Genetics, Department of Biomedicine, University of Basel, Basel, CH-4058 Switzerland; 99 Department of Genetics, Harvard Medical School, Boston, Massachusetts, Massachusetts 02115 USA; 100 Section of Neonatal Screening and Hormones, Department of Clinical Biochemistry, Immunology and Genetics, Statens Serum Institut, Copenhagen, DK-2300 Denmark; 101 Department of Psychiatry, Fujita Health University School of Medicine, Toyoake, Aichi 470-1192 Japan; 102 Regional Centre for Clinical Research in Psychosis, Department of Psychiatry, Stavanger University Hospital, 4011 Stavanger, Norway; 103 Rheumatology Research Group, Vall d’Hebron Research Institute, Barcelona, 08035 Spain; 104 Centre for Medical Research, The University of Western Australia, Perth, WA6009 Australia; 105 The Perkins Institute for Medical Research, The University of Western Australia, Perth, WA6009 Australia; 106 Department of Medical Genetics, Medical University, Sofia, 1431 Bulgaria; 107 Department of Psychology, University of Colorado Boulder, Boulder, Colorado 80309 USA; 108 Campbell Family Mental Health Research Institute, Centre for Addiction and Mental Health, Toronto, Ontario M5T 1R8 Canada; 109 Department of Psychiatry, University of Toronto, Toronto, Ontario M5T 1R8 Canada; 110 Institute of Medical Science, University of Toronto, Toronto, Ontario M5S 1A8 Canada; 111 Institute of Molecular Genetics, Russian Academy of Sciences, Moscow, 123182 Russia; 112 Latvian Biomedical Research and Study Centre, Riga, LV-1067 Latvia; 113 Department of Psychiatry and Zilkha Neurogenetics Institute, Keck School of Medicine at University of Southern California, Los Angeles, California 90089 USA; 114 Faculty of Medicine, Vilnius University, LT-01513 Vilnius, Lithuania; 115 Department of Biology and Medical Genetics, 2nd Faculty of Medicine and University Hospital Motol, 150 06 Prague, Czech Republic; 116 Department of Child and Adolescent Psychiatry, Pierre and Marie Curie Faculty of Medicine, Paris, 75013 France; 117 Duke-NUS Graduate Medical School, Singapore, 169857 Singapore; 118 Department of Psychiatry, Hadassah-Hebrew University Medical Center, Jerusalem, 91120 Israel; 119 Centre for Genomic Sciences, The University of Hong Kong, Hong Kong, China; 120 Mental Health Centre and Psychiatric Laboratory, West China Hospital, Sichuan University, Chengdu, 610041 Sichuan China; 121 Department of Biostatistics, Johns Hopkins University Bloomberg School of Public Health, Baltimore, Maryland 21205 USA; 122 Department of Psychiatry, Columbia University, New York New York, 10032 USA; 123 Priority Centre for Translational Neuroscience and Mental Health, University of Newcastle, Newcastle, NSW2300 Australia; 124 Department of Genetics and Pathology, International Hereditary Cancer Center, Pomeranian Medical University in Szczecin, 70-453 Szczecin, Poland; 125 Department of Mental Health and Substance Abuse Services, National Institute for Health and Welfare, PO BOX30, FI-00271 Helsinki, Finland; 126 Department of Mental Health, Bloomberg School of Public Health, Johns Hopkins University, Baltimore, Maryland 21205 USA; 127 Department of Psychiatry, University of Bonn, D-53127 Bonn, Germany; 128 Centre National de la Recherche Scientifique, Laboratoire de Génétique Moléculaire de la Neurotransmission et des Processus Neurodégénératifs, Hôpital de la Pitié Salpêtrière, 75013 Paris, France; 129 Department of Genomics Mathematics, University of Bonn, D-53127 Bonn, Germany; 130 Research Unit, Sørlandet Hospital, 4604 Kristiansand, Norway; 131 Department of Psychiatry, Harvard Medical School, Boston, Massachusetts 02115 USA; 132 VA Boston Health Care System, Brockton, Massachusetts 02301 USA; 133 Department of Psychiatry, National University of Ireland Galway, Co Galway, Ireland; 134 Centre for Cognitive Ageing and Cognitive Epidemiology, University of Edinburgh, Edinburgh, EH16 4SB UK; 135 Division of Psychiatry, University of Edinburgh, Edinburgh, EH16 4SB UK; 136 Division of Mental Health and Addiction, Oslo University Hospital, 0424 Oslo, Norway; 137 Massachusetts Mental Health Center Public Psychiatry Division of the Beth Israel Deaconess Medical Center, Boston, Massachusetts 02114 USA; 138 Estonian Genome Center, University of Tartu, Tartu, 50090 Estonia; 139 School of Psychology, University of Newcastle, Newcastle, NSW 2308 Australia; 140 First Psychiatric Clinic, Medical University, Sofia, 1431 Bulgaria; 141 Department P, Aarhus University Hospital, DK-8240 Risskov, Denmark; 142 Department of Psychiatry, Royal College of Surgeons in Ireland, Dublin 2, Ireland; 143 King’s College London, London, SE5 8AF UK; 144 Maastricht University Medical Centre, South Limburg Mental Health Research and Teaching Network, EURON, 6229 HX Maastricht, The Netherlands; 145 Institute of Translational Medicine, University of Liverpool, Liverpool, L69 3BX UK; 146 Max Planck Institute of Psychiatry, 80336 Munich, Germany; 147 Munich Cluster for SystemsNeurology (SyNergy), 80336 Munich, Germany; 148 Department of Psychiatry and Psychotherapy, Jena University Hospital, 07743 Jena, Germany; 149 Department of Psychiatry, Queensland Brain Institute and Queensland Centre for Mental Health Research, University of Queensland, Brisbane, Queensland, St Lucia QLD 4072 Australia; 150 Department of Psychiatry and Behavioral Sciences, Johns Hopkins University School of Medicine, Baltimore, Maryland 21205 USA; 151 Department of Psychiatry, Trinity College Dublin, Dublin 2, Ireland; 152 Eli Lilly and Company, Lilly Corporate Center, Indianapolis, 46285 Indiana USA; 153 Department of Clinical Sciences, Psychiatry, Umeå University, SE-901 87 Umeå, Sweden; 154 DETECT Early Intervention Service for Psychosis, Blackrock, Co Dublin Ireland; 155 Centre for Public Health, Institute of Clinical Sciences, Queen’s University Belfast, Belfast, BT12 6AB UK; 156 Lawrence Berkeley National Laboratory, University of California at Berkeley, Berkeley, California 94720 USA; 157 Institute of Psychiatry, Kings College London, London, SE5 8AF UK; 158 Melbourne Neuropsychiatry Centre, University of Melbourne & Melbourne Health, Melbourne, Vic 3053 Australia; 159 Department of Psychiatry, University of Helsinki, PO Box 590, FI-00029 HUS Helsinki, Finland; 160 Public Health Genomics Unit, National Institute for Health and Welfare, PO BOX 30, FI-00271 Helsinki, Finland; 161 Medical Faculty, University of Belgrade, 11000 Belgrade, Serbia; 162 Department of Psychiatry, University of North Carolina, Chapel Hill, North Carolina 27599-7160 USA; 163 Institute for Molecular Medicine Finland, FIMM, University of Helsinki, PO Box 20, FI-00014 Helsinki, Finland; 164 Department of Epidemiology, Harvard School of Public Health, Boston, Massachusetts 02115 USA; 165 Department of Psychiatry, University of Oxford, Oxford, OX3 7JX UK; 166 Virginia Institute for Psychiatric and Behavioral Genetics, Virginia Commonwealth University, Richmond, Virginia 23298 USA; 167 Institute for Multiscale Biology, Icahn School of Medicine at Mount Sinai, New York, New York 10029 USA; 168 PharmaTherapeutics Clinical Research, Pfizer Worldwide Research and Development, Cambridge, Massachusetts 02139 USA; 169 Department of Psychiatry and Psychotherapy, University of Gottingen, 37073 Göttingen, Germany; 170 Psychiatry and Psychotherapy Clinic, University of Erlangen, 91054 Erlangen, Germany; 171 Hunter New England Health Service, Newcastle, NSW 2308 Australia; 172 School of Biomedical Sciences, University of Newcastle, Newcastle, NSW 2308 Australia; 173 Division of Cancer Epidemiology and Genetics, National Cancer Institute, Bethesda, Maryland 20892 USA; 174 University of Iceland, Landspitali, National University Hospital, 101 Reykjavik, Iceland; 175 Department of Psychiatry and Drug Addiction, Tbilisi State Medical University (TSMU), N33, 0177 Tbilisi, Georgia; 176 Research and Development, Bronx Veterans Affairs Medical Center, New York New York, 10468 USA; 177 Wellcome Trust Centre for Human Genetics, Oxford, OX3 7BN UK; 178 deCODE Genetics, 101 Reykjavik, Iceland; 179 Department of Clinical Neurology, Medical University of Vienna, 1090 Wien, Austria; 180 Lieber Institute for Brain Development, Baltimore, Maryland 21205 USA; 181 Department of Medical Genetics, University Medical Centre Utrecht, Universiteitsweg 100, 3584 CG Utrecht, The Netherlands; 182 Berkshire Healthcare NHS Foundation Trust, Bracknell, RG12 1BQ UK; 183 Section of Psychiatry, University of Verona, 37134 Verona, Italy; 184 Department of Psychiatry, University of Oulu, PO Box 5000, Oulu, 90014 Finland; 185 University Hospital of Oulu, PO Box 20, 90029 OYS Oulu, Finland; 186 Molecular and Cellular Therapeutics, Royal College of Surgeons in Ireland, Dublin 2, Ireland; 187 Health Research Board, Dublin 2, Ireland; 188 School of Psychiatry and Clinical Neurosciences, The University of Western Australia, Perth, WA6009 Australia; 189 Computational Sciences CoE, Pfizer Worldwide Research and Development, Cambridge, Massachusetts 02139 USA; 190 Human Genetics, Genome Institute of Singapore, A STAR, Singapore, 138672 Singapore; 191 University College London, London, WC1E 6BT UK; 192 Department of Neuroscience, Icahn School of Medicine at Mount Sinai, New York New York, 10029 USA; 193 Institute of Neuroscience and Medicine (INM-1), Research Center Juelich, 52428 Juelich, Germany; 194 Department of Genetics, The Hebrew University of Jerusalem, 91905 Jerusalem, Israel; 195 Neuroscience Discovery and Translational Area, Pharma Research and Early Development, F Hoffman-La Roche, CH-4070 Basel, Switzerland; 196 Centre for Clinical Research in Neuropsychiatry, School of Psychiatry and Clinical Neurosciences, The University of Western Australia, Medical Research Foundation Building, Perth, WA6000 Australia; 197 Virginia Institute for Psychiatric and Behavioral Genetics, Departments of Psychiatry and Human and Molecular Genetics, Virginia Commonwealth University, Richmond, Virginia 23298 USA; 198 The Feinstein Institute for Medical Research, Manhasset, New York 11030 USA; 199 The Hofstra NS-LIJ School of Medicine, Hempstead, New York 11549 USA; 200 The Zucker Hillside Hospital, Glen Oaks, New York 11004 USA; 201 Saw Swee Hock School of Public Health, National University of Singapore, Singapore, 117597 Singapore; 202 Queensland Centre for Mental Health Research, University of Queensland, Brisbane, 4076 Queensland Australia; 203 Center for Human Genetic Research and Department of Psychiatry, Massachusetts General Hospital, Boston, Massachusetts 02114 USA; 204 Department of Child and Adolescent Psychiatry, Erasmus University Medical Centre, Rotterdam, 3000 The Netherlands; 205 Department of Complex Trait Genetics, Neuroscience Campus Amsterdam, VU University Medical Center Amsterdam, Amsterdam, 1081 The Netherlands; 206 Department of Functional Genomics, Center for Neurogenomics and Cognitive Research, Neuroscience Campus Amsterdam, VU University, Amsterdam, 1081 The Netherlands; 207 University of Aberdeen, Institute of Medical Sciences, Aberdeen, AB25 2ZD UK; 208 Departments of Psychiatry, Neurology, Neuroscience and Institute of Genetic Medicine, Johns Hopkins School of Medicine, Baltimore, Maryland 21205 USA; 209 Department of Clinical Medicine, University of Copenhagen, Copenhagen, 2200 Denmark

## Abstract

Previous studies have shown an increased risk for mental health problems in children born to both younger and older parents compared to children of average-aged parents. We previously used a novel design to reveal a latent mechanism of genetic association between schizophrenia and age at first birth in women (AFB). Here, we use independent data from the UK Biobank (N = 38,892) to replicate the finding of an association between predicted genetic risk of schizophrenia and AFB in women, and to estimate the genetic correlation between schizophrenia and AFB in women stratified into younger and older groups. We find evidence for an association between predicted genetic risk of schizophrenia and AFB in women (P-value = 1.12E-05), and we show genetic heterogeneity between younger and older AFB groups (P-value = 3.45E-03). The genetic correlation between schizophrenia and AFB in the younger AFB group is −0.16 (SE = 0.04) while that between schizophrenia and AFB in the older AFB group is 0.14 (SE = 0.08). Our results suggest that early, and perhaps also late, age at first birth in women is associated with increased genetic risk for schizophrenia in the UK Biobank sample. These findings contribute new insights into factors contributing to the complex bio-social risk architecture underpinning the association between parental age and offspring mental health.

## Introduction

**Figure 1:**
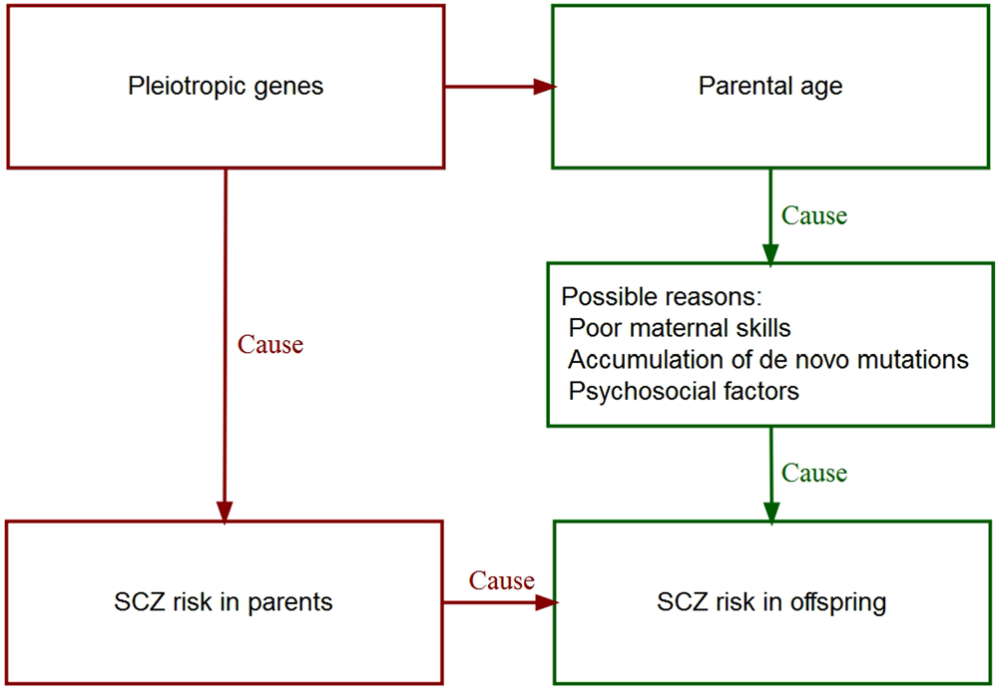
A flowchart of suggested mechanisms contributing to the relationship between the parental age and the schizophrenia risk in offspring.

An increased risk for a range of mental health issues in children born to both younger and older parents compared to children of average-aged parents has been reported in many studies^1–8^, with a particular focus on the risk of schizophrenia (SCZ) in children associated with parental age^9–12^. A recent comprehensive analysis using family data extracted from the Danish Psychiatric Central Register reported a relationship between mother’s age and risk of SCZ in her offspring^13^. They showed that there was higher risk in children of younger and older mothers compared to those of intermediate age (25–29 years – i.e. a U-shaped relationship between maternal age and risk of SCZ in offspring), but it was unclear if this was due to psychosocial factors associated with maternal age or if mothers at higher genetic risk for SCZ tend to have children at an earlier or later age. Moreover, the very high correlation in spousal ages makes paternal and maternal contributions to this relationship difficult to disentangle. A number of possible latent mechanisms behind these epidemiological observations have been proposed^14^, including shared genetic risk factors between parents and offspring^15,16^ (Fig. 1). A better understanding of factors contributing to the relationship between parental age and risk of psychiatric disorders is important for informing any future public health initiatives targeting this relationship.

We have previously reported evidence for a genetic relationship between maternal age at first birth (AFB) and risk of SCZ^17^, as illustrated in red in Fig. 1. We employed a novel design that directly tests the genetic risk of SCZ in mothers depending on AFB. In all previous study designs the psychiatric disorder was measured in the child, and hence the relationship with AFB in the mother was confounded with characteristics of the father. Here, and in our previous study we examine the relationship between SCZ and AFB by using a genetic predictor of SCZ in the mother. The analyses use community samples of women enrolled in research studies that were not enriched for psychiatric disorders (<1% for diagnosis with SCZ). The genetic predictor for SCZ can be calculated for all women in the studies as a function (such as weighted sum) of the SCZ risk alleles they carry, with the risk alleles identified as having increased frequencies in SCZ cases compared to controls. A woman’s genetic risk for schizophrenia is solely a function of her DNA, which she received independently of the characteristics of her partner. Hence, a benefit of this novel design is that the inferred association is not confounded by artefactual or non-genetic association(s) such as increased SCZ risk in offspring due to maternal environmental effects or by confounding with father’s age. We showed that the U-shaped relationship (between maternal age at birth and SCZ risk in offspring) observed in epidemiological studies was also observed when considering predicted genetic risk for SCZ as a function of AFB in healthy women^13,17^.

In this study, we replicate and extend our earlier findings^17^ using the UK Biobank data in which community samples of women have been measured for AFB. First, we confirm that SCZ polygenic risk score (PRS) for women in the UK Biobank with a record of AFB (N = 38,892) significantly predicts the U-shaped relationship found in McGrath *et al*.^13^, thereby replicating the results in Mehta *et al*.^17^. Second, we test if there is a genetic heterogeneity for AFB between younger and older AFB groups. Third, we estimate the genetic correlation between SCZ and AFB in younger and older AFB groups.

## Results

### Overview

In total, 41,630 SCZ GWAS samples including 18,957 cases and 22,673 controls from 30 cohorts were used in this study (Table S1), which were the same data used in Mehta *et al*.^17^. For the UK Biobank sample, 38,892 women were used. The distribution of AFB, age at interview, and year of birth for the UK Biobank data after QC are shown in Fig. S1. In total, 518,992 SNPs passed the quality control criteria and were in common across the SCZ and UK Biobank samples. The distribution of MAF is shown in Fig. S2. Figure S3 shows that there were no closely related individuals in the UK Biobank and SCZ case-control data sets, confirming that the two data sets were independent.

We estimated SCZ PRS for each individual in the UK Biobank sample, using the SCZ GWAS as a reference data set (see Methods). We used both the genetic profile score approach^18^ (PRS-score) and genomic best linear unbiased prediction method (PRS-GBLUP). We assessed the U-shaped relationship between AFB and SCZ PRS for the UK Biobank sample. We emphasize that in this novel design, it was not necessary to measure SCZ risk in offspring, and in our strategy potential confounding due to a correlation between paternal and maternal age was mostly removed.

Subsequently, we estimated SNP-heritability and genetic correlation between AFB and SCZ. Because of the non-linear relationship (U-shape), we divided the UK Biobank sample into two groups with younger and older AFB. We assessed if the younger and older AFB groups were genetically heterogeneous, and if there is any significant genetic correlation between SCZ and each of the younger and older AFB groups.

### Relationship between SCZ PRS and AFB

**Figure 2:**
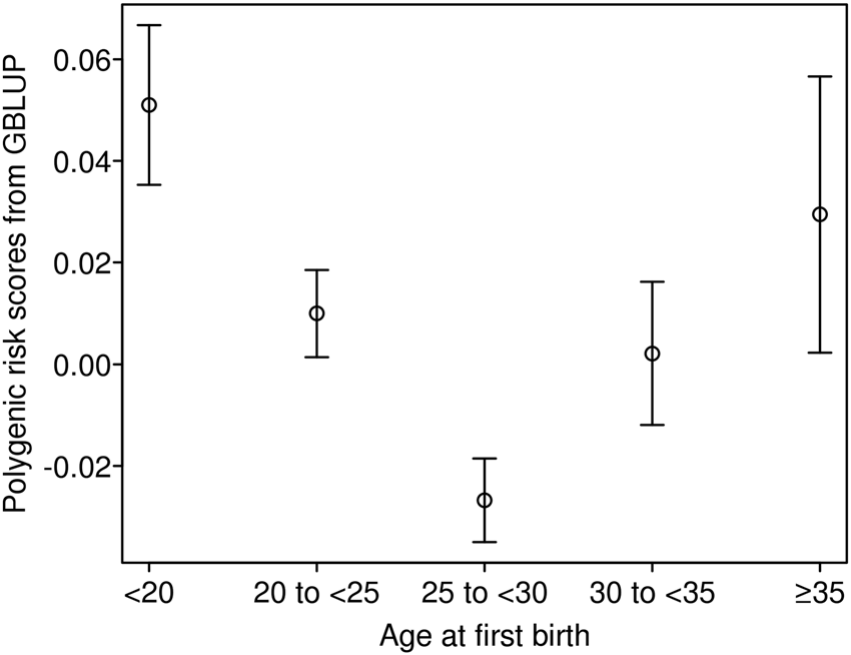
Mean and standard error of schizophrenia polygenic risk scores estimated from Genomic Best Linear Unbiased Prediction (GBLUP) in the UK Biobank sample grouped by age at first birth.

Consistent with McGrath *et al*.^13^ and replicating the findings in Mehta *et al*.^17^, a U-shaped relationship was observed between AFB and SCZ PRS-GBLUP (Fig. 2 and Table S2), implying genetic pleiotropic effects on AFB and SCZ risk. Figure 2 shows the mean and standard error of SCZ PRS-GBLUP in the UK Biobank sample grouped by AFB. The mean SCZ PRS-GBLUP in women with early AFB (<20 years) was significantly higher than that in women with intermediate AFB (P-value = 2.2E-02 for AFB between 20 to <25 years, P-value = 1.2E-05 for AFB between 25 to <30 years, P-value = 2.0E-02 for AFB between 30 to <35 years, in Table S3), but not in women with high AFB (P-value = 4.9E-01 for AFB ≥ 35 years). The mean SCZ PRS-GBLUP in women with AFB between 25 to <30 years was significantly lower than that in women with AFB between 20 to <25 years (P-value = 2.0E-03). Our results confirmed the findings in Mehta *et al*.^17^, i.e. a U-shaped relationship between AFB and SCZ PRS attributed to latent genetic factors, with stronger significance. The results were similar whether PRS was calculated using GBLUP (PRS-GBLUP) or conventional profile scoring based on GWAS summary statistics from the SCZ GWAS data (PRS-score) (Fig. S4 and Table S3). We also confirmed that the U-shaped relationship was replicated with estimated SNP effects from the full PGC SCZ GWAS study (PRS-scorePGC) (See Fig. S4 and Table S4), although these results could be biased due to possible sample overlap or the presence of relatives between the UK Biobank and the full PGC SCZ data.

### Linear predictor

**Figure 3:**
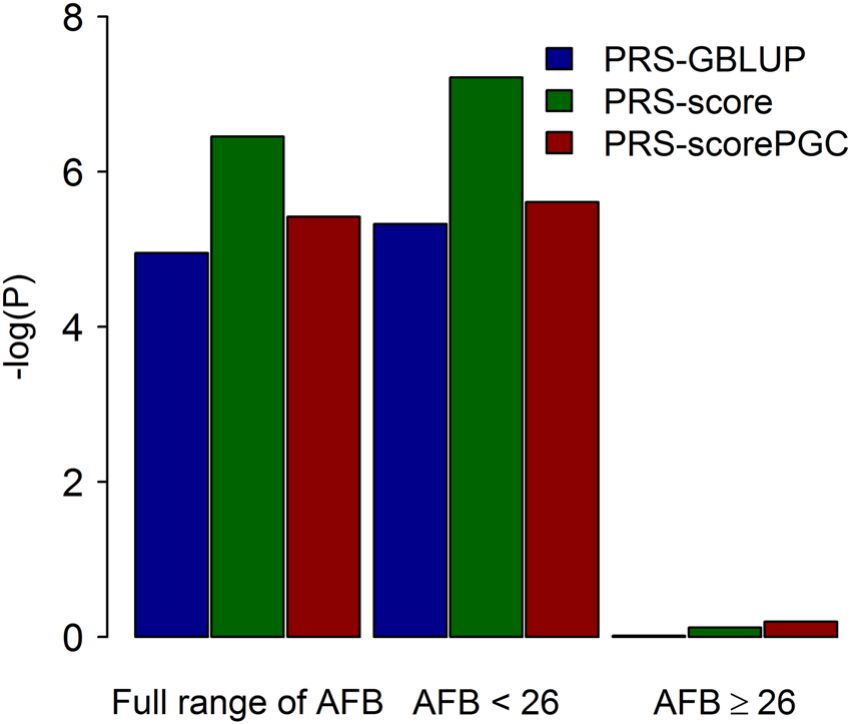
−log(P) values for the null hypothesis of R^2^ = 0 based on the linear prediction. Full range of AFB: All available samples with a record of age at first birth were used. AFB < 26 (≥26): Analyses were only focus on samples with AFB < 26 (≥26). PRS-GBLUP: Schizophrenia (SCZ) polygenic risk scores estimated from genomic best linear unbiased prediction were used as an explanatory variable in the model. PRS-score: SCZ polygenic risk scores estimated from genome-wide association study based on available individual genotype data were used as an explanatory variable in the model. PRS-scorePGC: SCZ polygenic risk scores estimated from summary statistics results of full PGC SCZ GWAS study were used as an explanatory variable in the model. Response variables were generated with a polynomial function derived by Mehta *et al*.^17^, which describes the relationship between SCZ risk in offspring and maternal age (z = 2.7214 − 0.1105X + 0.0018X^2^, where X is age at first birth), and used in the model in which the AFB phenotypes were adjusted for age at interview, year of birth, assessment center at which participant consented, genotype batch, and the first 20 principal components.

Following Mehta *et al*.^17^, we tested if SCZ PRS could predict the response variable (see Methods) that described the relationship between SCZ risk in offspring and maternal age derived in McGrath *et al*.^13^. Figure 3 shows that the response variable was significantly predicted by SCZ PRS for the group with the full range of AFB (P-value = 1.12E-05 for PRS-GBLUP, and P-value = 3.53E-07 for PRS-score and P-value = 3.08E-06 for PRS-scorePGC) and the subgroup with AFB younger than 26 (P-value = 4.71E-06, 6.06E-08 and 2.45E-06 for PRS-GBLUP, PRS-score and PRS-scorePGC, respectively), but not for the subgroup with AFB older than 26. The prediction with PRS-score was stronger than that with PRS-scorePGC, and both stronger than that with PRS-GBLUP although the results across the methods were not substantially different (Fig. 3).

Education level, income level, smoking and alcohol drinker status were additionally used to adjust the response variable in the linear prediction to test if those factors diminish the signals. Even with this conservative model, our results for the group with full range of AFB and the subgroup with AFB younger than 26 remained significant (Fig. S5 and Table S5), albeit with reduced effect size and significance. The reduced significance might be partly explained by the reduced sample size (i.e. for full range of AFB, N = 38,892 in the base model and 31,848 in model adjusted for education and income; see Table S5). In sensitivity analyses we also restricted the sample to those recruited at age ≥45 years (N = 35,451), which included the vast majority of women with a record of AFB, so that results were not biased by the exclusion of women with no reported AFB measure. We found that there was no substantive difference in our results despite the reduced sample size (Table S5 vs. S6).

The UK Biobank sample was divided into two subgroups born before or after 1945, a boundary of postponement of AFB based on the theory of the second democratic transition^19^. For individuals born after 1945, PRS-GBLUP significantly predicted the response variable for the group with the full range of AFB and the subgroup with AFB younger than 26, even after adjusting for socioeconomic status, and smoking and alcohol drinker status, but not for the subgroup with older AFB. For individuals born before 1945, PRS-GBLUP did not significantly predict the response variable for the group with the full range of AFB (P-value = 6.52E-02) nor the subgroup with AFB younger or older than 26 (P-value = 4.99E-02 or 4.38E-01) (Table S7). Our results agreed with the results of Mehta *et al*.^17^ in that SCZ PRS of women significantly predicted the response variable for the group with the full range of AFB and the subgroup with AFB younger than 26. The signals became stronger for the individuals born after year 1945.

### Genetic correlation between AFB and SCZ

Given that the AFB for women in the UK Biobank data was significantly predicted by PRS-GBLUP (P-value = 1.12E-05 and R^2^ = 4.96E-04 in Table S5), it was of interest to estimate the genetic correlation between AFB and SCZ, which is the scaled proportion of variance that AFB and SCZ share due to genetic factors.

**Figure 4:**
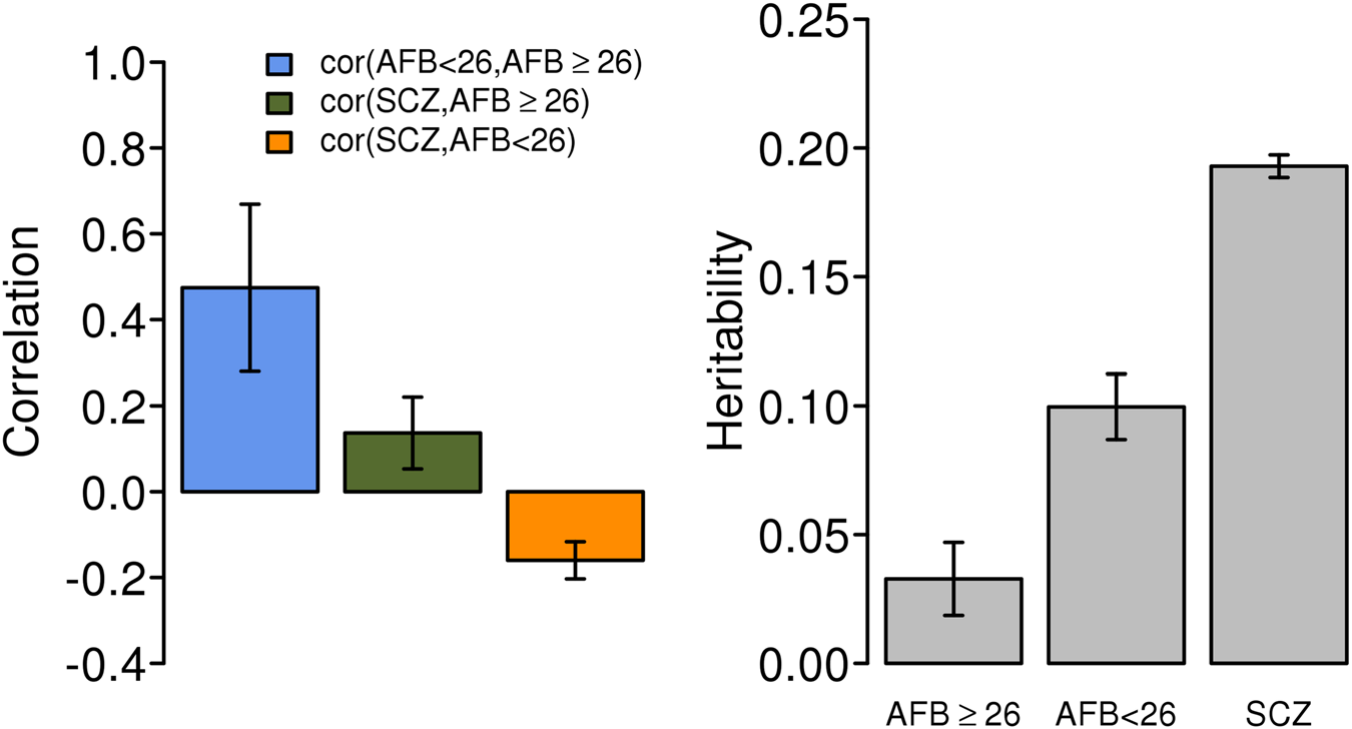
Genetic correlation (left) and heritability (right) of age at first birth (AFB) ≥ 26, AFB < 26, and schizophrenia (SCZ). Cor(AFB < 26, AFB ≥ 26): Estimated genetic correlation between the groups with AFB < 26 and with AFB ≥ 26. Cor(SCZ, AFB ≥ 26): Estimated genetic correlation between SCZ and AFB in the older AFB group. Cor(SCZ, AFB < 26): Estimated genetic correlation between SCZ and AFB in the younger AFB group. The bars are standard errors. In the model, the AFB phenotypes were adjusted for age at interview, year of birth, assessment center at which participant consented, genotype batch and the first 20 principal components. And the SCZ phenotypes were adjusted for sex, cohorts and the first 20 principal components. The sample size for group AFB ≥ 26 was 17,598 and for group AFB < 26 was 21,294, and for group SCZ was 41,630.

The SNP-heritability was 0.03 (SE = 0.01), 0.10 (SE = 0.01), and 0.20 (SE = 0.004), for older AFB, younger AFB, and SCZ, respectively. The SNP-heritability for the older AFB group was not significantly different from 0 (Fig. 4 right panel).

The estimated genetic correlation between younger and older AFB groups was significantly less than 1 (r_g_ = 0.47, SE = 0.19, P-value = 3.45E-03, in Table S8), indicating that younger and older AFB were genetically heterogeneous in the UK Biobank (Fig. 4 left panel). We demonstrate that a truncated selection had little impact on the estimation of the genetic correlation^20^ (Supplemental Notes 1 and 2 and Figs S6 and S7). Further, the estimated genetic correlation between SCZ and AFB in the younger AFB group was −0.16 (SE = 0.04) and that between SCZ and AFB in the older AFB group was 0.14 (SE = 0.08) (Fig. 4 left panel).

In sensitivity analyses, education level, income level, smoking and alcohol drinker status were additionally used to adjust the phenotypes in the genomic residual maximum likelihood (GREML) analyses to see if those factors change the estimates. Figure S8 shows that the estimates and their significance were slightly reduced, which could be partly explained by the decrease of sample size (N = 38,892 in the base model versus 31,848 in the model adjusted for education and income; see Table S8).

In addition to GREML, linkage disequilibrium score regression (LDSC)^4,21^ was applied to estimate the genetic correlation between SCZ and younger and older AFB (Table S9). As recommended by the LDSC papers^4,21^, pre-estimated LD scores from the 1000 Genome reference sample without constraining the intercept of regression were applied to the QCed GWAS data and full PGC SCZ GWAS summary results. However, we could access individual genotype data and it was clearly known that there was no overlapping sample and no high relatedness in the QCed GWAS data for which we would be able to use LD scores estimated based on the actual genotype data as well as to constrain the intercept as zero. As reported^4,21^, it was observed from simulations (Supplemental Note 3) that if there was no overlapping sample, LDSC with constraining the intercept as zero gave the most accurate estimate with the least standard error (Fig. S9). In the real data analyses, Table S9 showed that LDSC with constraining the intercept as zero gave similar estimates and standard errors, compared to those from GREML when using the QCed data. When explicitly estimating the intercept, the standard errors became large; therefore the precision of estimates might be decreased (Table S9). When using the full PGC SCZ GWAS summary, LDSC gave similar estimates but larger standard errors, compared to GREML estimates, although a larger SCZ sample size was used for LDSC analyses (77,096 for LDSC vs. 41,630 for GREML).

## Discussion

Parental age has been consistently associated with an increased risk of SCZ in offspring^9–13,15,16,22,23^, but it is well known that traditional epidemiological study designs, based on data measured for parental age and SCZ status in their offspring, have limitations with respect to disentangling genetic effects from non-genetic confounding effects such as common and residual environmental effects (Fig. 1). The elevated risk of SCZ associated with parental age extends to children of both younger and older parents, compared to children of average aged parents – i.e. a U-shaped relationship^13^. The most widely accepted mechanism, in the case of delayed parenthood, is a causal relationship due to the accumulation of *de novo* mutations with age (e.g. Kong *et al*.^24^), although this cannot explain increased risk in offspring of younger parents. This hypothesis is biologically plausible^25–28^, but Gratten *et al*.^29^ have shown using theory and simulations that paternal age-related *de novo* mutations are unlikely to be the major causal factor responsible for increased risk of SCZ in offspring. Instead, they argued that increased risk of SCZ in offspring of older fathers could be due to genetic overlap between risk of SCZ and delayed parenthood. This finding is consistent with epidemiological studies showing that paternal age at first child, as opposed to paternal age at conception, accounts for the increased risk of SCZ in the children of older fathers (i.e. arguing against a direct causal role for age-related *de novo* mutations)^15,16^. Notably, this mechanism of genetic overlap between parental age and SCZ applies equally well to offspring SCZ risk associated with early parenthood.

Recently, Mehta *et al*.^17^ used a novel design to investigate the genetic relationship between SCZ and AFB in women that is free of many of the potential confounders present in epidemiological study designs (e.g. poor maternity skill, psychosocial factors and shared environmental factors). Specifically, they used SNP effects obtained using SCZ case-control data to estimate genetic risk of SCZ in a general community sample of women measured for AFB. As expected, psychiatric disorder including SCZ was not enriched in this community sample (i.e. a general population). Therefore, there is hardly phenotypic assortative mating for the psychiatric disorder in the sample. In addition, the analyses were restricted to individuals with pairwise genomic relationship <0.05 across samples to make sure that SCZ GWAS and AFB samples were independent. Thus, it was unlikely that they shared the common environmental factors. In this analysis, we used the novel design and replicated the finding in a much larger and independent community sample, the UK Biobank study. We confirmed the U-shaped relationship between AFB and SCZ PRS reported by Mehta *et al*.^17^ (Figs 2 and 3), and provided evidence of genetic overlap between SCZ and AFB in women. The large number of samples in the UK Biobank made it possible to also estimate genetic variance and covariance between SCZ and AFB using a linear mixed model, showing that the traits of younger and older AFB are genetically heterogeneous (Fig. 4 or Table S8). The genetic correlation between SCZ and AFB in women with AFB < 26 was −0.16 (SE = 0.04), which was significantly different from zero (Fig. 4). The genetic correlation between SCZ and AFB in women with AFB ≥ 26 was 0.14 (SE = 0.08), which was significantly larger, albeit by a marginal amount, than zero in a one-tail Wald test (p-value = 0.04). This shows that there is at least a suggestive signal for the genetic correlation between older AFB and SCZ, hence we cannot totally rule out the possibility of the association.

In linear prediction, results from PRS-score were similar to or more significant than those from PRS-scorePGC (Table S5). This is noteworthy because PRS-scorePGC was based on the GWAS summary statistics from the full PGC SCZ GWAS (33,640 cases, 43,456 controls, from 52 cohorts), which is a larger sample than that used to generate PRS-score (individual-level genotype data on 18,957 cases, 22,673 controls, from 30 cohorts). However, publicly available GWAS summary statistics such as those used for PRS-scorePGC provide incomplete information about sample overlap or pairwise relationships between data sets, either of which could introduce biases or influence statistical significance due to non-independence of samples. There is an approach or strategy for relatedness QC without accessing individual genotypes^30^, however, it is not immediately applicable to the full PGC SCZ data. We hypothesize that the superior performance of PRS-score in our analysis, despite smaller sample size than PRS-scorePGC (which is explained by restrictions on data access for individual-level genotype data), reflects the very stringent QC we applied to the data, including on relatedness.

In this study, we could not test the significance of association between SCZ PRS and SCZ status for the UK Biobank sample because there was no information about SCZ outcome for the sample. However, we conducted an association test between the SCZ PRS and bipolar disorder (that is highly correlated with SCZ) for the UK Biobank (P-value = 1.02E-08 with 4,508 bipolar cases and 8,821 controls). This showed that the SCZ PRS was a reliable predictor of the true underlying SCZ liability for the UK Biobank sample. We also did not adjust for the assortative mating effects because the partner information is not available. However, it is not likely that such genetic assortative mating effects are substantial although a further study is required to test this hypothesis.

Estimated genetic correlations and their standard errors based on GREML and LDSC were very similar (Table S9), but only when the intercept of the LDSC was constrained, which requires the strong assumption of no overlapping samples, and only when LD scores were calculated from the actual sample, which is usually not possible when using GWAS summary statistics. We also observed this phenomenon in our results based on simulated data (Supplemental Note 3 and Fig. S9). These observations are in line with the statement in Bulik-Sullivan *et al*.^21^ that standard errors are sacrificed to achieve unbiased genetic correlation and the availability of individual-level genotype data was the most preferable scenario^31^. Nevertheless, the estimated genetic correlations between SCZ, younger AFB and older AFB were consistent using two different approaches, i.e. GREML and LDSC (Table S9).

In summary, this study replicated previously reported evidence for significant genetic overlap between risk of SCZ and AFB in women using an independent target sample from the UK Biobank. We further showed that AFB in women is genetically heterogeneous (comparing younger to older AFB) and that there is a significant genetic correlation between SCZ and AFB. Conducting parallel analyses for AFB in men is of great interest but these data are less easily available and AFB has not been recorded for men in the UK Biobank. Our results suggest that early, and perhaps also late, age at first birth in women is associated with increased genetic risk for SCZ in the UK Biobank samples. These findings contribute new insights into factors contributing to the complex bio-social risk architecture underpinning the association between parental age and offspring mental health.

## Methods and Materials

Informed consent was obtained from every participant in each study. Research Ethics approval was obtained from University of South Australia Human Research Ethics Committee (HREC). All methods were carried out in accordance with the relevant guidelines and regulations.

### Participants and quality control

#### Schizophrenia (SCZ) sample

Genome-wide association data were available from 18,987 SCZ cases and 22,673 controls from the second phase of the Psychiatric Genomics Consortium (PGC)^32^ with quality control (QC) applied as described in Mehta *et al*.^17^. In summary, before imputation, PGC group performed a QC for the raw genotypes within each cohort: SNPs with call rate <0.95 or Hardy-Weinberg equilibrium p-value < 10E-6 in controls or p-value < 10E-10 in cases were excluded. Individuals with call rate <0.98 were excluded. Then, the QCed raw genotype data were imputed with IMPUTE2/SHAPEIT^33,34^ using the full 1000 Genomes Project dataset^35^ as the reference set. Post-imputation quality control was performed in each cohort as: A best-guess genotype was called if its posterior probability >0.8, otherwise treated as missing. SNPs with an imputation r-squared <0.1 or MAF < 0.005, or call rate <0.98 were excluded. Out of 39 cohorts, eight cohorts were excluded because the number of SNPs passing the QC process was too small and one cohort was excluded because essential covariate information was not available. After the post-imputation QC, we combined the genotype data across all cohorts^17,32^. As in Mehta *et al*.^17^, we used HapMap3 SNPs that were reliable in estimating (shared) genetic architecture between complex traits^36–38^. In the combined dataset, SNPs with call rate <0.9, individuals with call rate <0.9 were excluded, and one individual in a pair with genomic relationship >0.05 was excluded. This less stringent QC for call rate was because SNP and individual QC had been already done by the PGC. After this QC, 688,145 SNPs (best-guess genotypes) and 41,630 individuals were remained and used to combine with the UK Biobank sample.

#### UK Biobank sample

In the first version of UK Biobank^39^ data set, 80,702 female (54,215 with a recode of AFB) out of 152,249 genotyped individuals were available from a community sample, in which psychiatric disorders were not enriched, with 72,355,667 imputed SNPs available. Out of all imputed SNPs, 1,242,190 HapMap3 SNPs were identified, which were filtered through the following QC filtering criteria: SNPs with imputation INFO < 0.6^17^, minor allele frequency (MAF) <0.01, call rate <0.95, and Hardy-Weinberg equilibrium P-value < 10–7^17^ were excluded. In addition, ambiguous strand SNPs were excluded. After this QC, 930,841 SNPs (best guess genotypes) remained and they were used to merge with the SCZ genotypic data. For individual level QC, only Caucasian females were used who clustered within 6 standard deviation from the mean of the EUR reference sample^40^ for the first and second genetic relationship principal components. Individuals were further excluded due to call rate <0.95. In addition, one in a pair of individuals was randomly removed if their genomic relationship coefficient was more than 0.05^17^. An important reason for removing closely related individuals was to reduce the possibility that the similarity between the phenotypes of those individuals could be caused by non-genetic effects (e.g. common environment effects)^41^. Furthermore, UK Biobank samples were excluded if their genomic relationship with any individual in the SCZ or AFB datasets used in Mehta *et al*.^17^ was >0.05, in order to ensure the independence of the UK Biobank sample. The AFB sample in Mehta *et al*.^17^ included 12,247 genotyped women measured for AFB, who were from four cohorts: Estonia, the Netherlands, Sweden, and the United Kingdom. After QC, we used 80,522 individuals (18,957 SCZ cases, 22,673 SCZ controls, and 38,892 UK Biobank individuals) and 518,992 SNPs in the main analyses.

### Statistical analyses

#### Estimation of SCZ polygenic risk score (PRS) in UK Biobank sample

We used a GBLUP model to generate SCZ PRS for each individual in the UK Biobank sample accounting for the genetic relationship between the UK Biobank sample and the SCZ case-control sample. The GBLUP model can be written as

$${\bf{y}}={\bf{X}}{\bf{b}}+{\bf{Z}}{\bf{u}}+{\bf{e}},$$

where **y** is a vector of phenotypic data (i.e. **1**s for SCZ cases, **0**s for SCZ controls and missing for UK Biobank individuals), **b** represents vectors of fixed effects including sex, cohort and 20 ancestry principal components (PCs)^42^, **u** is the vector of SCZ PRS, and **e** is the vector of residuals. **X** and **Z** are design matrices to allocate effects to phenotypic data. It is assumed that **u** is normally distributed as $N(0,\,{\bf{G}}{\sigma }_{u}^{2})$, where **G** is the genomic relationship matrix constructed as described in Yang *et al*.^43^ and ${\sigma }_{u}^{2}$ is the additive genetic variance, and **e** is normally distributed as $N(0,\,{\bf{I}}{\sigma }_{e}^{2})$, where **I** is an identify matrix and ${\sigma }_{e}^{2}$ is the residual variance. GBLUP was performed using GCTA^43^ or MTG2^44,45^ so that a subset of **u** for the UK Biobank sample could be inferred based on the phenotypes of the SCZ sample and the genomic relationships between the two data sets. Mean SCZ PRS in the UK Biobank individuals grouped by their AFB was estimated to assess the previously reported U-shaped relationship^13,17^. GBLUP provides more accurate PRS (PRS-GBLUP) under a polygenic genetic architecture than the more standard genetic profile score approach^18^, but for comparison we also calculated PRS by the standard method (PRS-score). To estimate SCZ risk SNP effects, an association test was conducted with PLINK 1.9^46^ using the same SCZ GWAS data used in the GBLUP analyses, with phenotypes adjusted for sex, cohort and 20 PCs. PRS-scores for individuals in the UK Biobank sample were generated by summing the count of SCZ risk alleles weighted by the SNP effects estimated from the association test. In addition to PRS-score, we used the estimated SNP effects from the full PGC SCZ GWAS study (33,640 cases and 43,456 controls; publicly available at https://www.med.unc.edu/pgc/)^32^ to calculate a further profile risk score in the UK Biobank sample (PRS-scorePGC). However, we cannot exclude the possibility of sample overlap or relatedness between the UK Biobank and full PGC SCZ data, because we only have a permission to access to the genotypes of 30 cohorts out of 52. Note that we used all of the SNPs across the genome (after QC) to calculate SCZ PRS for the UK biobank sample.

#### Linear prediction

It is of interest to replicate the findings from the epidemiological observation^13^ that maternal ages are associated with SCZ risk in offspring, using the novel design consisting of general community sample (UK Biobank) measured for AFB and SCZ PRS. Any significant association between AFB and SCZ PRS from a linear prediction gives evidence that the U-shape relationship between maternal ages and risk in offspring (observed in McGrath *et al*.^13^) can be explained by genetic factors. Using 2,894,688 records from the National Danish Registry, McGrath *et al*.^13^ reported a U-shaped relationship between risk of SCZ in children and maternal age at birth. The resulting equation from the U-shaped relationship (z = 2.7214 − 0.1105X + 0.0018X^2^) can be applied to data of age at first birth of women to generate predictors of risk of SCZ in their children^17^. We did not consider the second model in Mehta *et al*.^17^ that was adjusted for partner’s ages because the model was shown to be over-corrected^17^. We calculated the response variable (z) in the UK Biobank sample from the recorded age at first birth (X) and this was used as the y-variable in analyses regressing on either PRS-GBLUP, PRS-score or PRS-scorePGC and including age at interview, assessment center at which participant consented, genotype batch, year of birth and the first 20 PCs as covariates. Socioeconomic status (i.e. education and income level)^47^ or smoking and alcohol drinker status were additionally used to adjust the response variable in sensitivity analyses (Fig. S1). Linear models were applied to the group with the full range of AFB records, the subgroup with AFB younger than 26 years (<26), and the subgroup with AFB at or older than 26 (≥26), respectively, where the value of 26 is the mean of AFB in the UK Biobank sample. From the model, the coefficient of determination (R^2^) and P-value against the null hypothesis (i.e. SCZ PRS of women is not a predictor of AFB) were estimated.

#### Genomic residual maximum likelihood (GREML)

Since previous studies^13,17^ found that younger and older AFB have reciprocal relationship with risk of SCZ (i.e. a U-shaped relationship), it is of interest to test if AFB in women is a genetically heterogeneous trait, for instance by estimating the genetic correlation between younger and older AFB groups. If the genetic correlation is significantly different from 1, it would imply that the causal variants and/or their effect sizes differ between younger and older AFB. Moreover, it would be interesting to estimate genetic correlations between SCZ case-control data and younger or older AFB groups. The UK Biobank data were divided into two groups by younger (<26) and older AFB (≥26). Then, three-variate linear mixed model analysis was conducted to estimate genetic variance and covariance between SCZ case-control data, and younger and older AFB groups. The model can be written as

$$\begin{array}{ll}{{\bf{y}}}_{1}={{\bf{X}}}_{1}{{\bf{b}}}_{1}+{{\bf{Z}}}_{1}{{\bf{g}}}_{1}+{{\bf{e}}}_{1} & {\rm{for}}\,{\rm{SCZ}}\,{\rm{sample}}\\ {{\bf{y}}}_{2}={{\bf{X}}}_{2}{{\bf{b}}}_{2}+{{\bf{Z}}}_{2}{{\bf{g}}}_{2}+{{\bf{e}}}_{2} & {\rm{for}}\,{\rm{UK}}\,{\rm{Biobank}}\,{\rm{sample}}\,{\rm{with}}\,{\rm{AFB}} < 26\\ {{\bf{y}}}_{3}={{\bf{X}}}_{3}{{\bf{b}}}_{3}+{{\bf{Z}}}_{3}{{\bf{g}}}_{3}+{{\bf{e}}}_{3} & {\rm{for}}\,{\rm{UK}}\,{\rm{Biobank}}\,{\rm{sample}}\,{\rm{with}}\,{\rm{AFB}}\ge 26\end{array}$$

where **y** are three column vectors of phenotypic observations (one for each data set or group, i.e. SCZ case-control data set, UK Biobank AFB < 26 and UK Biobank AFB ≥ 26). For SCZ case-control data, pre-adjusted phenotypes corrected for sex, cohort and 20 PCs were used. For the UK Biobank sample, the AFB phenotypes were pre-adjusted for sex, age at interview, year of birth, assessment center, genotype batch and 20 PCs. Again, other possible confounding factors such as socioeconomic status, and smoking and alcohol drinker status were additionally controlled in sensitivity analyses to check if the estimates were substantially changed. The GREML analyses were conducted with GCTA^43^ or MTG2^44,45^ to estimate pairwise genetic correlations among the three data sets; SCZ data, and younger AFB and older AFB. We tested if the genetic correlation between SCZ and younger or older AFB was significantly different from zero. We also tested if genetic correlation between younger and older AFB was significantly different from 1 to assess heterogeneity between the two groups^48^. We used a Wald test to obtain p-values for the test.

## Electronic supplementary material

Supplementary file
