# Supplementary material for: Age at first birth in women is genetically associated with increased risk of schizophrenia
Source: Sci Rep. 2018 Jul 5;8:10168. doi: 10.1038/s41598-018-28160-z (PMC6033923; doi:10.1038/s41598-018-28160-z)
Supplement: Supplementary file 1 — Supplementary file [file 41598_2018_28160_MOESM1_ESM.pdf]

Supplemental Data

Age at first birth in women is genetically associated with increased risk of schizophrenia

Guiyan Ni, Jacob Gratten, Schizophrenia Working Group of the Psychiatric Genomics Consortium, Naomi R. Wray, and Sang Hong Lee

Figure

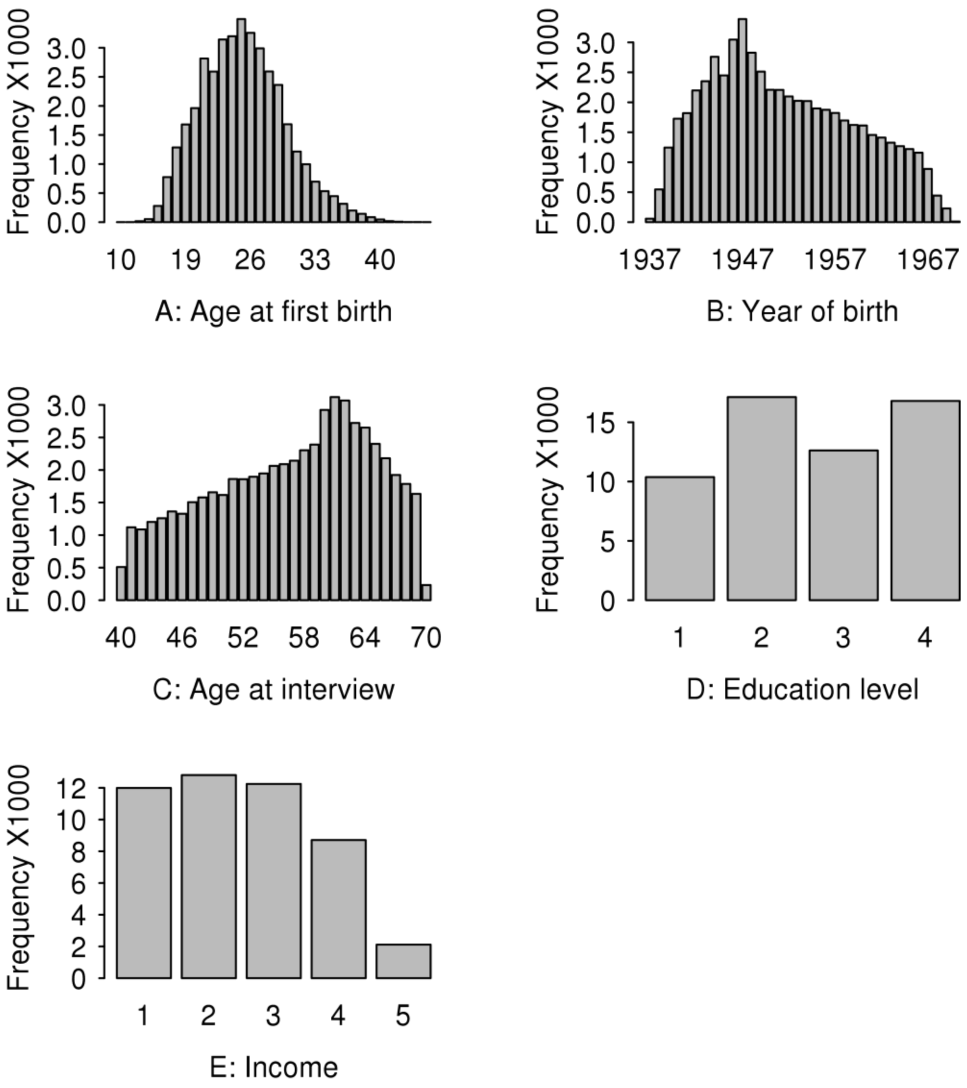

Figure S1. Summary of data after quality control.

A) Age at first birth. **B)** Year of birth. **C)** Age at interview. **D)** Education level: Based on Guggenheim et al.[1], the original 7-grouped education levels were categorized as 4 levels, i.e. (1) None, (2) O-levels or CSEs, (3) A-levels, NVQ, HND, HNC or other professional qualification and (4) College or University degree, **E)** Income: The averaged total household income before tax, categorized as 5 levels, i.e. (1) less than £18,000, (2) from £18,000 to £30,999, (3) from £31,000 to £51,999, (4) from £52,000 to £100,000 and (5) greater than £100,000.

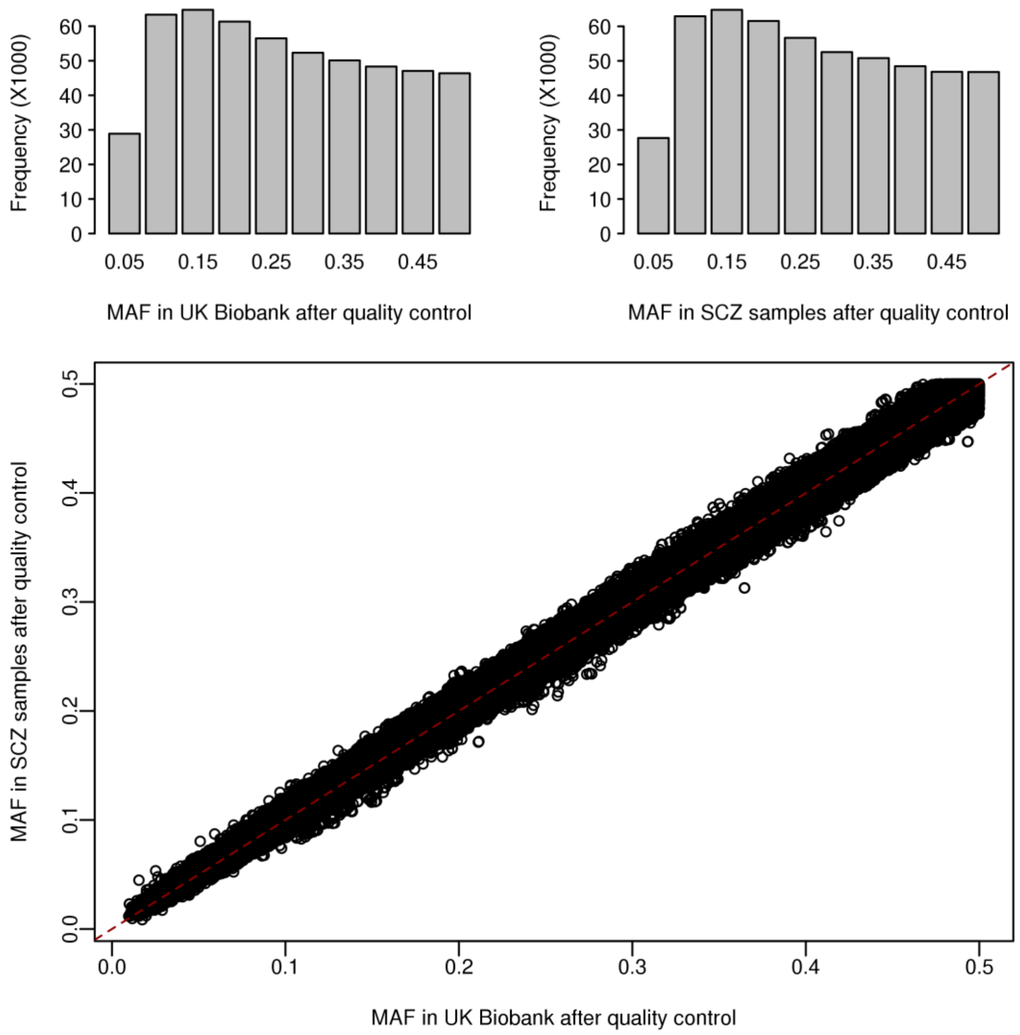

Figure S2. MAF of SNPs in UK Biobank and SCZ sample after quality control.
The number of SNPs was 518,992.

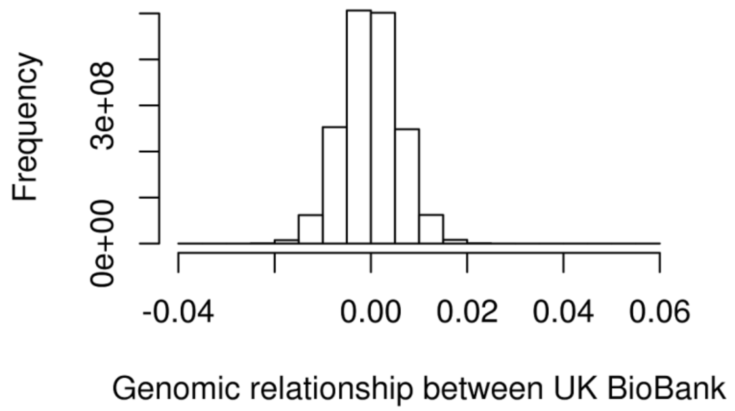

Figure S3. The off-diagonal entries of genomic relationship matrix based on the quality controlled UK Biobank and SCZ sample.

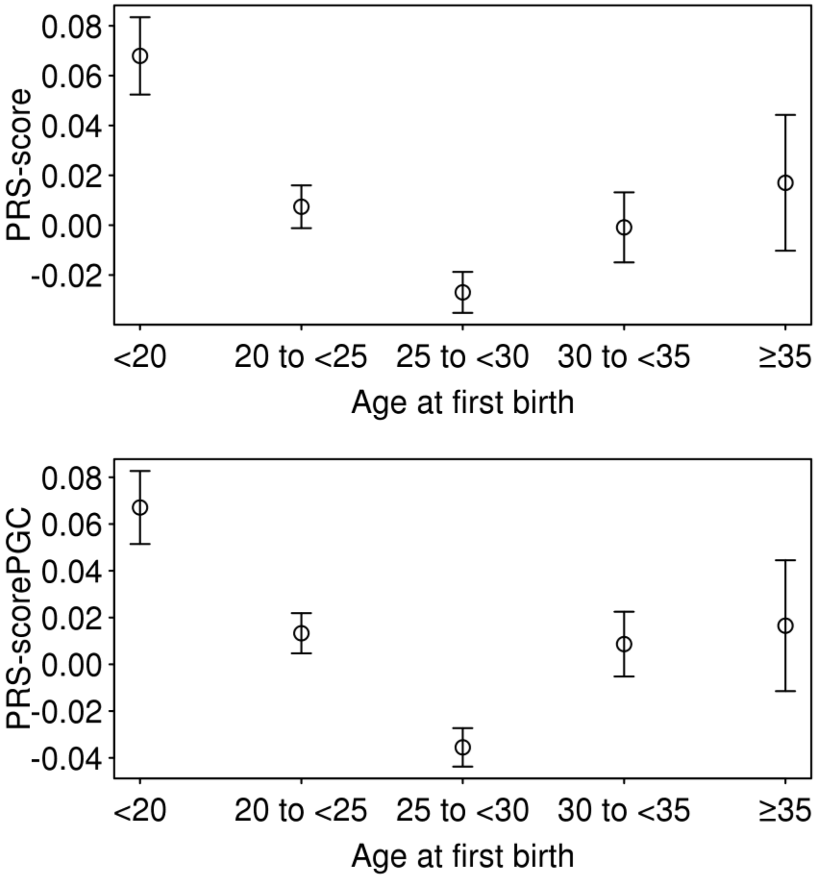

Figure S4. Means and standard errors of schizophrenia PRS-score (top panel) and PRS-scorePGC (bottom panel) in the UK Biobank sample grouped by age at first birth.
PRS-scores were estimated from the GWAS summary statistics that were performed based on the current QCed genotype data of SCZ sample. PRS-scorePGC were estimated from the publicly available GWAS summary statistics from the full PGC SCZ GWAS[2].

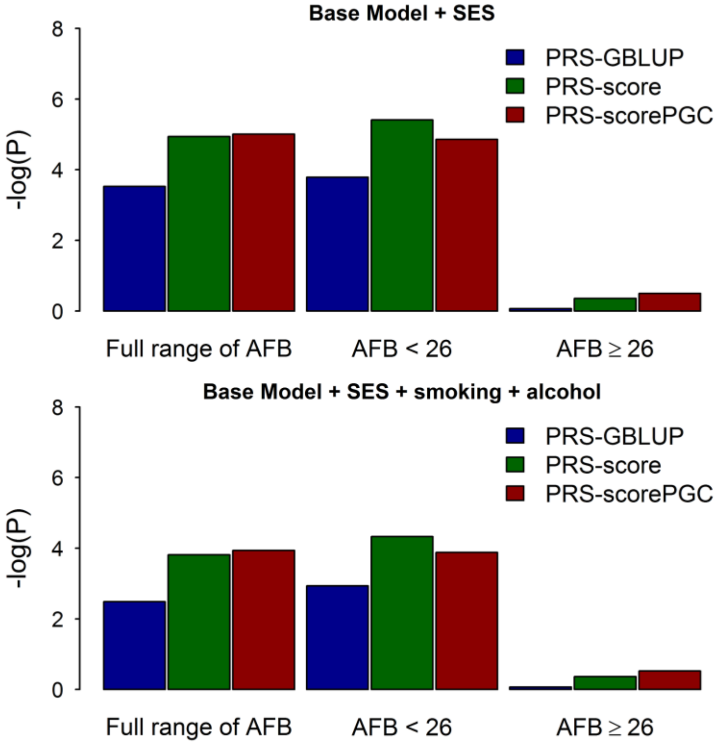

Figure S5. -log(P) values under the null hypothesis of $R^2 = 0$ based on a linear model using different samples.

Full range of AFB: All available samples with a record of age at first birth were used. AFB < 26 (≥26): Analyses were only focused on samples with AFB < 26 (≥26). Base Model: The AFB phenotypes were adjusted for age at interview, year of birth, assessment center at which the participant consented, genotype batch, and the first 20 principal components. Base Model + SES: The AFB phenotypes were adjusted for socioeconomic status (i.e. education and income level), in addition to the factors adjusted in the base model. Base Model + SES + smoking + alcohol: The AFB phenotypes were also adjusted for smoking and alcohol drinking status in addition to the Base model + SES. PRS-GBLUP: Schizophrenia (SCZ) polygenic risk scores estimated from genomic best linear unbiased prediction were used as an explanatory variable in the model. PRS-score: SCZ polygenic risk scores estimated from GWAS based on the available individual genotype data were used as an explanatory variable in the model. PRS-scorePGC: SCZ polygenic risk scores estimated from the publicly available summary statistics of the full PGC SCZ GWAS were used as an explanatory variable in the model. Response variables were generated with a polynomial function derived by Mehta et al.[3], which describes the relationship between SCZ risk in offspring and maternal age ($z = 2.7214 - 0.1105X + 0.0018X^2$, where X is age at first birth), and used in the model.

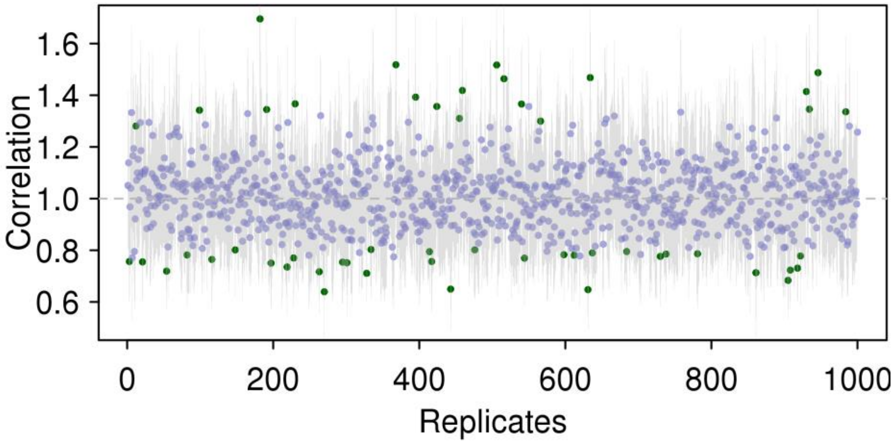

Figure S6. Means and 95% confidence intervals of genetic correlation in simulated data. The dark green dots (N=52) are estimated genetic correlations that are significantly different from 1 in 1000 replicates.

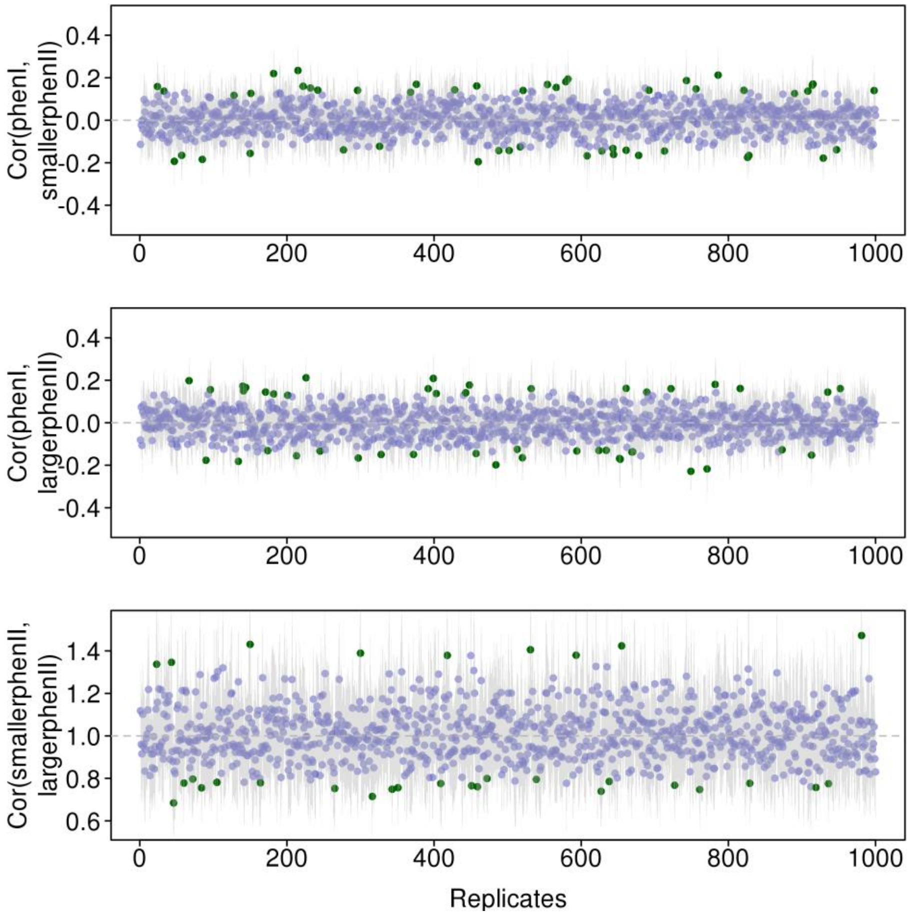

Figure S7. Means and 95% confidence intervals of genetic correlation in simulated data.
The dark green dots (N=50, 45 and 32 for the upper, middle and lower panel) are estimated genetic correlations that are significantly different from 0 (for the upper and middle panel) or 1 (for the bottom panel). Each panel shows results from 1000 replicates.

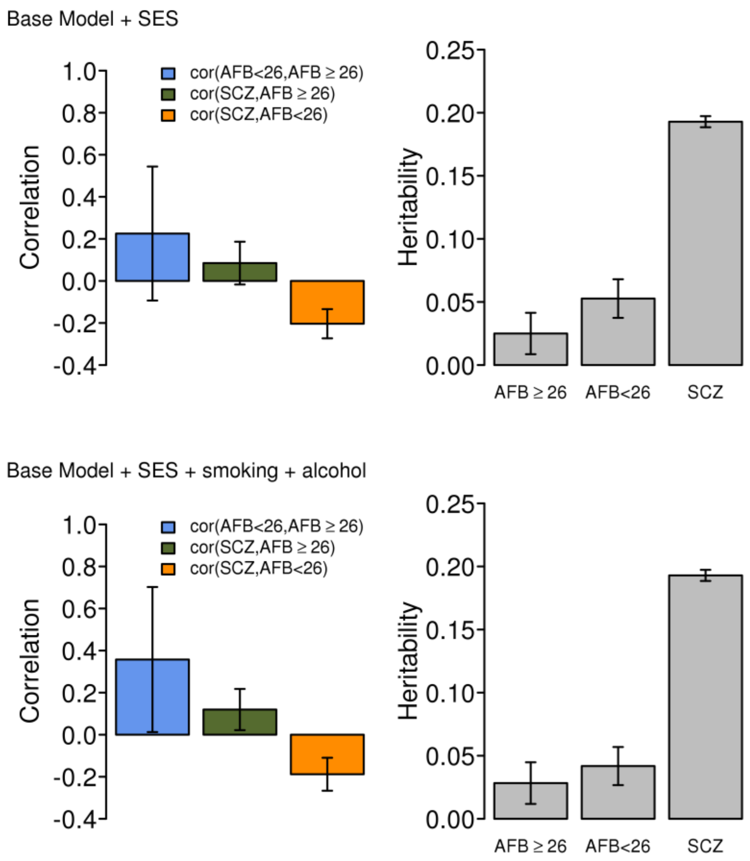

Figure S8. Genetic correlation (left) and heritability (right) of SCZ risk, age at first birth < 26, and age at first birth ≥ 26 based on different models.

Base Model: The AFB phenotypes were adjusted for age at interview, year of birth, assessment center at which the participant consented, genotype batch, and the first 20 principal components. Base Model + SES: The AFB phenotypes were adjusted for socioeconomic status (i.e. education and income level), in addition to the factors adjusted in the base model. Base Model + SES + smoking + alcohol: The AFB phenotypes were also adjusted for smoking and alcohol drinking status in addition to the Base model + SES. Cor(AFB<26, AFB ≥26): Estimated genetic correlation between the groups with AFB < 26 and with AFB ≥ 26. Cor(SCZ, AFB≥26): Estimated genetic correlation between SCZ and AFB in the older AFB group. Cor(SCZ, AFB<26): Estimated genetic correlation between SCZ and AFB in the younger AFB group. In the top panel, sample sizes were 41630, 16838, and 15,010 for SCZ, younger and older AFB group, respectively. In the bottom panel, samples sizes were 41,630, 16,789, and 14,988 for SCZ, younger and older AFB group, respectively.

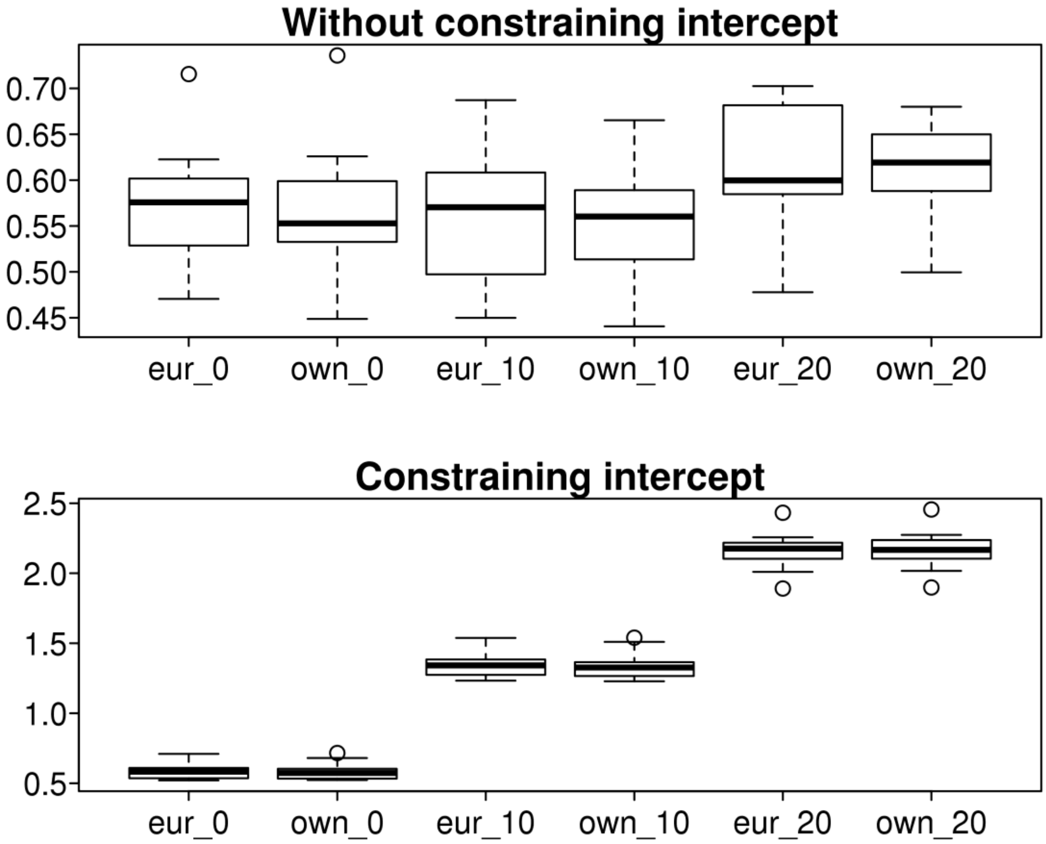

Figure S9. Genetic correlation estimated with LDSC from different scenarios based on simulated phenotype.
'Without constraining intercept': the intercept in LDSC was estimated from the data. 'Constraining intercept': the intercept was constrained as 0, assuming there is no overlapping samples in two data sets. Eur: the LD scores were estimated from European samples in 1000 Genome. Own: the LD scores were estimated from the available individual-level genotype data. The percentage of overlapping individuals was labeled as 0, 10 or 20 after 'eur' or 'own' symbol (e.g. eur_0 or own_0). The detailed simulation process is in Supplemental Note 3. The simulated true genetic correlation was 0.6. The method was considered as unbiased if the estimated genetic correlation was not significantly different from 0.6.

Table

Table S1. Number of individuals of the SCZ sample used in the current study[a]

Cohort	Case	Control	N	Cohort	Case	Control	N
ajsz	77	138	215	lacw	153	264	417
asrb	438	284	722	lemu	106	114	220
buls	188	597	785	lie2	122	251	373
butr	588	574	1162	lie5	460	354	814
cims	62	52	114	mgs2	2506	2575	5081
clm2	3330	4033	7363	msaf	151	73	224
clo3	2067	873	2940	pewb	549	1804	2353
cou3	522	539	1061	pews	148	229	377
denm	462	449	911	swe5	1586	2380	3966
dubl	250	801	1051	swe6	742	972	1714
edin	358	277	635	top8	376	397	773
egcu	171	857	1028	ucla	668	594	1262
ersw	233	294	527	uktr	25	30	55
gras	998	1129	2127	umeb	232	400	632
irwt	1279	986	2265	umes	111	353	464
**Total**	**18,957**	**22,673**	**41,630**				

[a]Not all of the cohorts from the PGC2 SCZ sample were publicly available and we had access to 39 out of 52 cohorts only. Out of 39 cohorts, eight cohorts were excluded because the number of SNPs passing the QC process was too small and one cohort was excluded because essential covariate information was not available.

Table S2. Mean and standard error of schizophrenia polygenic risk scores in the UK Biobank sample grouped by age at first birth

		< 20	20 to <25	25 to <30	30 to <35	≥35
PRS-GBLUP[a]	Estimate	0.051	0.010	-0.027	0.002	0.029
	SE	0.016	0.009	0.008	0.014	0.027
PRS-score[b]	Estimate	0.068	0.007	-0.027	-0.001	0.017
	SE	0.016	0.009	0.008	0.014	0.027
PRS-scorePGC[c]	Estimate	0.067	0.013	-0.036	0.009	0.017
	SE	0.016	0.009	0.008	0.014	0.028

[a]Polygenic risk scores calculated using GBLUP.
[b]Polygenic risk scores calculated using GWAS summary statistics from the SCZ GWAS data.
[c]Polygenic risk scores calculated using GWAS summary statistics from the full SCZ GWAS study.

Table S3. P-value of testing significant difference of PRS-GBLUP (lower triangle) and PRS-score (upper triangle) between AFB groups

	< 20	20 to <25	25 to <30	30 to <35	≥35
<20	1	6.42E-04	6.68E-08	1.01E-03	1.04E-01
20 to <25	2.2E-02	1	3.86E-03	6.15E-01	7.37E-01
25 to <30	1.2E-05	2.0E-03	1	1.09E-01	1.23E-01
30 to <35	2.0E-02	6.3E-01	7.6E-02	1	5.60E-01
≥35	4.9E-01	4.9E-01	4.8E-02	3.7E-01	1

PRS-GBLUP: schizophrenia (SCZ) polygenic risk scores estimated from genomic best linear unbiased prediction were used as an explanatory variable in the model. PRS-score: SCZ polygenic risk scores estimated from genome-wide association study based on the available individual genotype data were used as an explanatory variable in the model. Response variables were generated with a polynomial function derived by Mehta et al.[3], which describes the relationship between SCZ risk in offspring and maternal age ($z = 2.7214 - 0.1105X+0.0018X^2$, where X is age at first birth), and used in the model.

Table S4. P-value of testing significant difference of PRS-scorePGC[a] between AFB groups

	<20	20 to <25	25 to <30	30 to <35	≥35
<20	1				
20 to <25	2.56E-03	1			
25 to <30	6.44E-09	4.07E-05	1		
30 to <35	5.13E-03	7.76E-01	6.10E-03	1	
≥35	1.15E-01	9.12E-01	7.47E-02	8.01E-01	1

[a]PRS-scorePGC: SCZ polygenic risk scores estimated from publicly available summary statistics results of the full PGC SCZ GWAS study with 33,640 cases and 43,456 controls were used as an explanatory variable in the model. Response variables were generated with a polynomial function derived by Mehta et al.[3], which describes the relationship between SCZ risk in offspring and maternal age ($z = 2.7214 - 0.1105X+0.0018X^2$, where X is age at first birth), and used in the model.

Table S5. Coefficients of determination (R^2), P values and number of individuals used in different linear models based on different samples

			PRS-GBLUP		PRS-score[a]		PRS-scorePGC[b]	
		#individuals	R^2	P Value	R^2	P Value	R^2	P Value
Full range of AFB	Base model[c]	38892	4.96E-04	1.12E-05	6.67E-04	3.53E-07	5.49E-04	3.80E-06
	Base model + SES	31848	4.11E-04	2.98E-04	6.04E-04	1.15E-05	6.13E-04	9.92E-06
	Base model + SES + smoking + alcohol	31777	2.72E-04	3.26E-03	4.51E-04	1.53E-04	4.68E-04	1.15E-04
<26	Base model	21294	9.83E-04	4.71E-06	1.38E-03	6.06E-08	1.04E-03	2.46E-06
	Base model + SES	16838	8.43E-04	1.64E-04	1.27E-03	3.88E-06	1.12E-03	1.38E-05
	Base model + SES + smoking + alcohol	16789	6.27E-04	1.17E-03	9.87E-04	4.68E-05	8.70E-04	1.32E-04
>=26	Base model	17598	9.79E-08	9.67E-01	5.42E-06	7.58E-01	1.26E-05	6.37E-01
	Base model + SES	15010	2.17E-06	8.57E-01	3.97E-05	4.40E-01	6.70E-05	3.16E-01
	Base model + SES + smoking + alcohol	14988	2.27E-06	8.54E-01	4.16E-05	4.30E-01	7.19E-05	2.99E-01

[a]Estimated from GWAS based on the available genotype data.

[b]Estimated form the full PGC SCZ GWAS study with 33,640 cases and 43,456 controls, which are publicly available (https://www.med.unc.edu/pgc/).

[c]The AFB phenotypes were adjusted for age at interview, year of birth, assessment center at which participant consented, genotype batch, and the first 20 principal components.

Table S6. Coefficients of determination (R^2), P values and number of individuals used in different linear models based on samples with age at interview older than 45

		R^2	P Value	#individuals
PRS-GBLUP	Base model[a]	4.57E-04	5.65E-05	35451
	Base model + SES	3.63E-04	1.25E-03	28712
	Base model + SES+ smoking + alcohol	2.33E-04	9.75E-03	28643
PRS-score[b]	Base model	6.15E-04	3.03E-06	35451
	Base model + SES	5.08E-04	1.34E-04	28712
	Base model + SES+ smoking + alcohol	3.71E-04	1.12E-03	28643
PRS-scorePGC[c]	Base model	5.06E-04	2.28E-05	35451
	Base model + SES	5.28E-04	9.92E-05	28712
	Base model + SES + smoking + alcohol	4.09E-04	6.22E-04	28643

[a]The AFB phenotypes were adjusted for age at interview, year of birth, assessment center at which the participant consented, genotype batch, and the first 20 principal components.
[b]Estimated from GWAS based on the available genotype data.
[c]Estimated from the full SCZ GWAS study with 33,640 cases and 43,456 controls, which are publicly available (https://www.med.unc.edu/pgc/).

Table S7. Coefficients of determination (R^2), P values and number of individuals used in different linear models based on samples born before or after the year 1945

		Born after 1945			Born before 1945 (including 1945)		
		R^2	P Value	#individuals	R^2	P Value	#individuals
Full range of AFB	Base model	5.84E-04	6.40E-05	27365	2.95E-04	6.52E-02	11527
	Base model + SES	4.47E-04	1.26E-03	23278	1.97E-04	1.94E-01	8570
	Base model + SES + smoking + alcohol	2.88E-04	9.65E-03	23223	1.36E-04	2.80E-01	8554
AFB < 26	Base model	1.31E-03	1.95E-05	13872	5.18E-04	4.99E-02	7422
	Base model + SES	9.22E-04	1.18E-03	11418	6.11E-04	6.88E-02	5420
	Base model + SES+ smoking + alcohol	6.97E-04	4.87E-03	11379	4.60E-04	1.15E-01	5410
AFB ≥ 26	Base model	9.32E-06	7.23E-01	13493	1.47E-04	4.38E-01	4105
	Base model + SES	3.09E-06	8.48E-01	11860	2.82E-04	3.46E-01	3150
	Base model + SES + smoking + alcohol	4.66E-06	8.14E-01	11844	3.74E-04	2.78E-01	3144

Table S8. P-value testing if the genetic correlation between younger and older AFB is significantly different from 1, or that between AFB and SCZ is significantly different from 0

	Cor (AFB<26, AFB≥26)[a]	Cor (SCZ, AFB≥26)[b]	Cor (SCZ, AFB<26)[b]	#individuals
Base Model	3.45E-03	1.02E-01	2.22E-04	38892
Base model + SES	7.52E-03	4.04E-01	3.40E-03	31848
Base model + SES + smoking + alcohol	3.12E-02	2.22E-01	1.64E-02	31777

[a]Testing if genetic correlation is significantly different from 1.
[b]Testing if genetic correlation is significantly different from 0.

Table S9. Genetic correlation between younger, older AFB and SCZ estimated from LDSC and GREML

		Cor(AFB<26,AFB≥26)		Cor(SCZ,AFB<26)		Cor(SCZ,AFB≥26)	
Data	Method	Estimate	SE	Estimate	SE	Estimate	SE
QCed GWAS data (18,957 SCZ cases, 22,673 SCZ controls, 57,428 UK Biobank)	1000 Genome[a], no constrain[b]	0.674	0.3122	-0.2252	0.0878	-0.0039	0.1262
	1000 Genome, constrain[c]	0.5106	0.2066	-0.1516	0.0431	0.1236	0.0833
	Sample[d], no constrain	0.372	0.2724	-0.1552	0.0768	-0.0007	0.1231
	Sample, constrain	0.4915	0.2025	-0.1546	0.0422	0.1202	0.0793
Full GWAS summary results (33,640 SCZ cases, 43,456 SCZ controls, 57,428 UK Biobank)	1000 Genome, no constrain	-	-	-0.1235	0.0738	0.1471	0.112
QCed GWAS data	GREML	0.4743	0.1946	-0.1599	0.0433	0.1368	0.0837

[a]LD score were pre-computed LD Scores which were estimated based on European-ancestry individuals in 1000 Genome (https://data.broadinstitute.org/alkesgroup/LDSCORE/eur_w_ld_chr.tar.bz2).
[b]The LD Score regression intercept was estimated from data.
[c]The LD Score regression intercept was constrained as 0.
[d]Use current individual genotype data to estimate LD score.
The genetic correlation was estimated base on the Base Model where the AFB phenotypes were adjusted for age at interview, year of birth, assessment center at which participant consented, genotype batch, and the first 20 principal components.

Supplemental Note 1

Unbiased estimation of the genetic correlation between two groups with a truncated selection

Assuming that a random variable *y* is distributed as *N* (0, 1), a linear model can be written as

$y = g + e$

where *g* is random genetic effects, which are distributed as *N* (0, h^2), and *e* is random residuals, which are from *N* (0, 1-h^2). When the values for the phenotype *y* less than a threshold *t* are selected, the variables after the selection can be written as

$y_s = g_s + e_s.$

Following quantitative genetic theory[4], the mean and variance for the selected variable are

$E(y_s) = i,$

$E(y_s^2) = (1+it)$ and

$\mathrm{var}(y_s) = E(y_s^2) - E(y_s)^2 = (1+it) - i^2.$

The mean and variance for the genetic values after the selection are

$E(g_s) = E(y_s)h^2,$

$E(g_s^2) = h^2(1+h^2it)$ and

$$\mathrm{var}(g_s) = E(g_s^2) - E(g_s)^2 = h^2(1+h^2 i_2 t_2) - (h^2 i)^2. \quad (1)$$

The heritability after the selection is

$$h_s^2 = \frac{h^2[(1+h^2 i_2 t_2) - h^2 i^2]}{(1+it) - i^2}.$$

From Eq. (1), the genetic values after the selection can be defined as[5,6]

$$g_s = c + bg \quad (2)$$

where *c* is a constant and $b = \sqrt{\frac{(1+h^2 i_2 t_2) - h^2 i^2}{(1+it) - i^2}}$.

From Eq. (2), the genetic covariance between two sets of selected sample can be written as

$\mathrm{cov}(g_{s1}, g_{s2}) = b_1 b_2 \mathrm{cov}(g_1, g_2),$

and the genetic correlation is

$$\text{co}\,r(g_{s1}, g_{s2}) = \frac{b_1 b_2 \operatorname{cov}(g_1, g_2)}{b_1 \operatorname{var}(g_1) b_2 \operatorname{var}(g_2)} = cor(g_1, g_2). \qquad (3)$$

Therefore, from equation (3), it is clear that even when samples are ascertained with a truncated selection, the genetic correlation is unbiased, and there is no spurious estimation of heterogeneity.

Supplemental Note 2

In current study, the UK Biobank data were split into two groups according to their AFB measure, younger and older groups truncated by the mean of AFB (26 years). To test if the truncation data can bias the estimation of genetic correlation, a simulation was performed (simulation 1). The simulated phenotypes were divided into two groups (larger or smaller than the mean), and GREML was performed to estimate the genetic correlation. Over 1000 replicates, the average of the estimated genetic correlation was 1.02 (SE=0.004.2), and 52 out of 1000 replicates were significantly different from 1 (type I error, Supplementary Figure 6). Moreover, to mimic the estimation of the genetic correlation between SCZ and younger and older AFB, and to assess type I error rate under the null model, two phenotypes (phenI and phenII) were simulated such that the genetic correlation between two traits was zero in a second simulation (see simulation 2). Then, PhenII was divided into two groups (larger or smaller than mean). Afterwards, three-variate linear mixed model was used to estimate genetic variance and covariance between group with phenI, and groups with larger or smaller phenII. Over 1000 replicates, the estimated genetic correlation between phenI and group with smaller (or larger) phenII was 0.0047 ± 0.0021 (-0.001 ± 0.0021), and genetic correlation between smaller or larger phenII was 1 ± 0.0004. The type I error was 5% for all three estimated genetic correlations (Supplementary Figure 8). Overall, the simulated results showed that a truncated selection hardly biases the estimation of genetic correlation, which agreed with theory (Supplementary Note 1).

Simulation 1:

The simulation was conducted using MTG2 based on available genotyped data (section #10 in the manual)[7]. The genotype data of 20,000 out of 57,428 samples were randomly selected to be used in the simulation process. Out of 518,992 SNPs which passed the quality control criteria, 10,000 SNPs were randomly selected as QTLs and have effects drown from a normal distribution N (0, 1). True breeding values or genetic profile scores can be obtained from the product of SNP genotype coefficients and the corresponding SNP effects. The simulated phenotype can be generated as the sum of true breeding values and residual effects which follow a normal distribution N (0, 1). According to the phenotypic values, the data were split into two

groups (i.e. larger or smaller than the mean of simulated phenotype). Then, GREML was performed based on the real genotype data and simulate phenotype data to estimate the genetic correlation between the two groups. The number of replicates was 1000 each with a different set of QTLs and their effects.

Simulation 2:

The simulation was conducted with MTG2 [7] based on given genotyped data. The genotype data of 30,000 out of 80,522 samples (SCZ sample + UK Biobank sample) were randomly selected for the simulation process. Out of 518,992 SNPs which passed the quality control criteria, 10,000 SNPs were randomly selected as QTLs. In order to mimic SCZ and AFB, each QTL was assigned two independent effects which following a multivariate normal distribution. The covariance matrix of those two effects were $\begin{bmatrix} 1 & 0 \\ 0 & 1 \end{bmatrix}$ and the mean vector was [0, 0]. Thus, the genetic correlation between two simulated phenotypes (denoted as phenI and phenII) should be 0. True breeding values were obtained as the product of SNP genotype coefficients and the corresponding SNP effects. The simulated phenotype can be generated as the sum of true breeding values and residual effects which follow a multivariate normal distribution with mean [0, 0] and the covariance matrix $\begin{bmatrix} 1 & 0 \\ 0 & 1 \end{bmatrix}$. Among the 30,000 individuals, a random set of 10,000 individuals was made available for the first trait (phenI) only (but missing for the second trait). For the other 20,000 individuals whose phenotypes were available for the second trait (phenII) only (but, missing for the first trait), a truncated selection according to the second trait was applied to divide them into two groups (i.e. larger or smaller than the mean of phenII phenotypes). Three-variate linear mixed model was used to estimate genetic variance and covariance between the three groups. The number of replicates was 1000 each with a different set of QTLs and their effects. In this simulation, it would be expected that estimated genetic correlation between phenI and phenII was 0, and that between the groups with larger and smaller phenII phenotypes was 1.

Supplemental Note 3

Two sets of samples were selected, each with random 10,000 individuals, based on the real genotype data from the UK Biobank. The percentages of overlapped individuals were used as 0%, 10%, and 20%. Out of 518,992 SNPs, 10,000 SNPs were randomly selected as QTLs and assigned two SNP effects that were randomly drawn from a multivariate normal distribution MVN~$\left(\begin{bmatrix}0\\0\end{bmatrix}, \begin{bmatrix}1 & 0.6\\0.6 & 1\end{bmatrix}\right)$. True breeding values or genetic profile scores were obtained from the product of SNP genotype coefficients and the corresponding SNP effects. Residual effects were generated from MVN~$\left(\begin{bmatrix}0\\0\end{bmatrix}, \begin{bmatrix}1 & 0.8\\0.8 & 1\end{bmatrix}\right)$. Phenotype in each data set were generated as the sum of true breeding values and residual effects. Therefore, the heritabilites of two phenotypes were 0.5 for both data sets and genetic correlation between the two data sets was 0.6. The number of replicates was 20. For the LDSC analyses, GWAS were performed to obtain P values for each SNP in each data set. The LD scores used in the LDSC analyses were either obtained from European samples in 1000 Genomes or estimated from the current genotype data. The results based on this simulation are shown in Supplementary Figure 9.

List of abbrevations

AFB: Age at first birth

SCZ: Schizophrenia

GWAS: Genome-wide association study

GBLUP: Genomic best linear unbiased prediction

GREML: Genomic residual maximum likelihood

LDSC: Linkage disequilibrium score regression

PGC: Psychiatric genomics consortium

PRS: Polygenic risk score

PRS-score: PRS based on GWAS summary statistics from the SCZ GWAS data

PRS-GBLUP: PRS calculated using GBLUP

PRS-scorePGC: PRS from the full PGC SCZ GWAS study

PC: Principal component

QC: Quality control

MAF: Minor allele frequency

GCTA: Genome-wide Complex Trait Analysis

MTG2: Multi-Trait GREML and GBLUP

Supplemental References

1. Guggenheim, J.A., Williams, C., Eye, U.K.B. & Vision, C. Childhood febrile illness and the risk of myopia in UK Biobank participants. *Eye (London, England)* **30**, 608-14 (2016).
2. Schizophrenia Working Group of the Psychiatric Genomics Consortium. Biological insights from 108 schizophrenia-associated genetic loci. *Nature* **511**, 421-427 (2014).
3. Mehta, D. *et al.* Evidence for genetic overlap between schizophrenia and age at first birth in women. *JAMA Psychiatry* **73**, 497-505 (2016).
4. Falconer, D.S. & Mackay, T.F.C. *Introduction to Quantitative Genetics*, (Longmans Green, Harlow, Essex, UK, 1996).
5. Lee, S.H., Wray, N.R., Goddard, M.E. & Visscher, P.M. Estimating missing heritability for disease from genome-wide association studies. *American Journal of Human Genetics* **88**, 294-305 (2011).
6. Lee, S.H., Yang, J., Goddard, M.E., Visscher, P.M. & Wray, N.R. Estimation of pleiotropy between complex diseases using single-nucleotide polymorphism-derived genomic relationships and restricted maximum likelihood. *Bioinformatics* **28**, 2540-2542 (2012).
7. Lee, S.H. & van der Werf, J. MTG2: An efficient algorithm for multivariate linear mixed model analysis based on genomic information. *Bioinformatics* **32**, 1420-1422 (2016).
